# Expanded Ligands Based upon Iron(II) Coordination Compounds of Asymmetrical Bis(terpyridine) Domains

**DOI:** 10.3390/molecules28010082

**Published:** 2022-12-22

**Authors:** Dalila Rocco, Alessandro Prescimone, Catherine E. Housecroft, Edwin C. Constable

**Affiliations:** Department of Chemistry, University of Basel, BPR 1096, Mattenstrasse 24a, 4058 Basel, Switzerland

**Keywords:** 2,2′:6′,2″-terpyridine, 3,2′:6′,3″-terpyridine, 4,2′:6′,4″-terpyridine, iron(II), expanded ligand

## Abstract

The synthesis and characterization of two tritopic ligands containing a 2,2′:6′,2″-terpyridine (tpy) metal binding domain and either a 3,2′:6′,3″- or a 4,2′:6′,4″-tpy domain are detailed. The synthetic routes to these ligands involved the [Pd(dppf)Cl_2_]-catalyzed coupling of a boronic ester-functionalized 2,2′:6′,2″-tpy with bromo-derivatives of 3,2′:6′,3″-tpy or 4,2′:6′,4″-tpy. The 2,2′:6′,2″-tpy domains of the tritopic ligands preferentially bind Fe^2+^ in reactions with iron(II) salts leading to the formation of two homoleptic iron(II) complexes containing two peripheral 3,2′:6′,3″-tpy or 4,2′:6′,4″-tpy metal-binding sites, respectively. These iron(II) complexes are potentially tetratopic ligands and represent expanded versions of tetra(pyridin-4-yl)pyrazine.

## 1. Introduction

Coordination entities result from the binding of a metal centre to a ligand [1], and the metal centres may be mono- or multinuclear. A ligand is a chemical species with one or more electrons available to bind a metal, and the electrons may be present in ‘lone pairs’ or chemical bonds of the ligand [1]. Ligands are typically small-to-medium-sized organic or inorganic molecules, ions, or radicals, and the ligand may bind to the metal centres through one or more atoms. The latter case is described as chelating [1], and it is convenient to describe chelating ligands in terms of their metal-binding domains [2]. Typical chelating metal-binding domains include carboxylate (in an *O*,*O*’-bidentate binding mode), 1,3-diketonates, 2,2′-bipyridine, 1,10-phenanthroline and 2,2′:6′,2″-terpyridine.

A special case exists with ligands that possess additional metal-binding capacity after coordination to a metal centre. This additional capacity may be in a binding site that cannot coordinate to the same metal for steric or electronic reasons or, more rarely, when a potentially chelating ligand does not exhibit its maximum denticity. This latter situation is described as a hypodentate coordination mode [3]. A complex containing a ligand exhibiting a hypodentate bonding mode can act as a ligand itself. This new, metal-containing coordination unit has been described as a metalloligand [4,5,6,7,8,9].

**Scheme 1:**
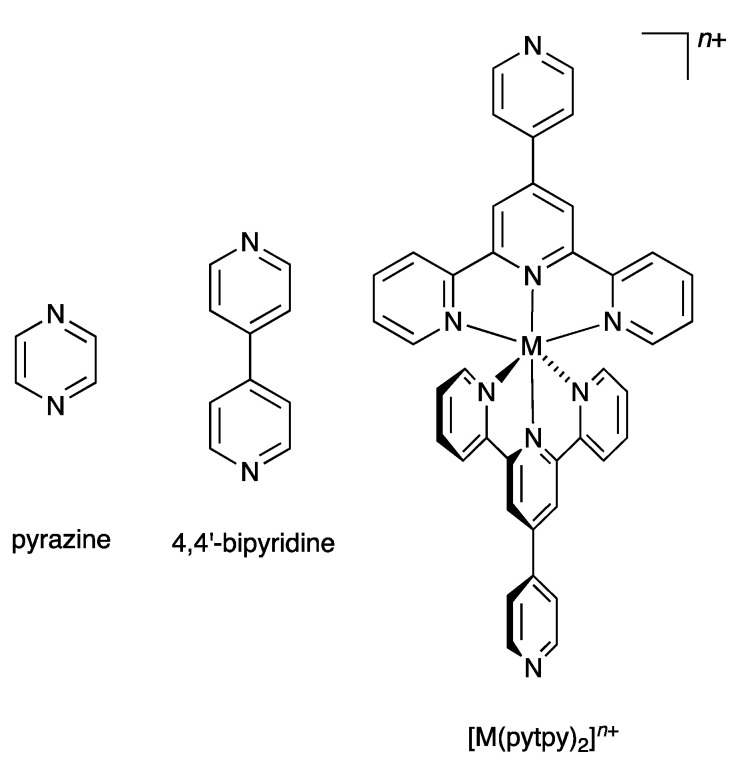
The concept of the “expanded ligand” illustrated with [M(pytpy)_2_]*^n^*^+^ as an expanded analogue of pyrazine or 4,4′-bipyridine.

**Scheme 2:**
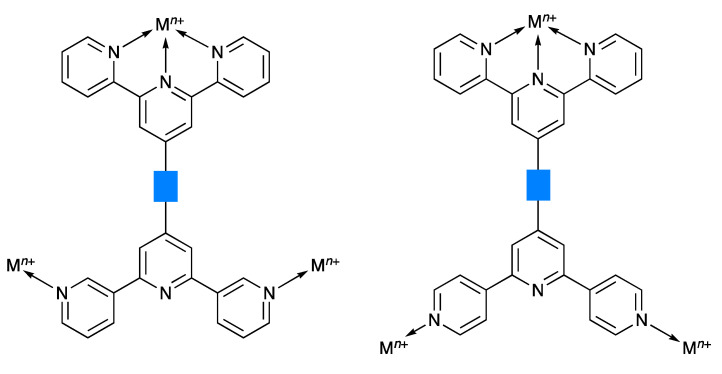
Schematic illustration of the coordination of tritopic ligands with 2,2′:6′,2″- and 3,2′:6′,3″-tpy or 2,2′:6′,2″- and 4,2′:6′,4″-tpy metal-binding units. The blue rectangle represents any organic spacer.

In 2007, we introduced the term “expanded ligand” to describe a class of compounds in which two or more metal-binding domains are separated by a metal-containing unit [10]. We have tended to use the term “expanded ligand” for metalloligands in which the central core involves classical chelating metal-binding domains, in particular oligopyridines [11,12,13,14,15,16,17,18]. The description “expanded ligand” emphasizes the relationship between the new coordination entity and the “parent” organic ligand. A typical example of an expanded ligand is a 4′-(pyridin-4-yl)-2,2′:6′,2″-terpyridine (pytpy) complex of the type [M(pytpy)_2_]*^n^*^+^ in which the central six-coordinate metal M is coordinated to two terdentate tpy metal-binding domains from each of two pytpy ligands. Each of the coordinated pytpy ligands has a nitrogen donor in the pyridin-4-yl available for further coordination. This [M(pytpy)_2_]*^n^*^+^ moiety can be regarded as an expanded pyrazine or an expanded 4,4′-bipyridine (Scheme 1). We have shown that this concept can usefully be used in the design of coordination networks involving pendant nitrogen and oxygen donors on oligopyridine scaffolds. We now report the synthesis of potentially tritopic ligands incorporating either 2,2′:6′,2″- and 3,2′:6′,3″-tpy or 2,2′:6′,2″- and 4,2′:6′,4″-tpy metal-binding domains (Scheme 2) and their iron(II) complexes which can be classified as expanded tetratopic ligands. We selected an {Fe(2,2′:6′,2″-tpy)_2_}^2+^ scaffold containing a low-spin d^6^ iron(II) centre for the preliminary investigation, as it permits parallel solution ^1^H NMR spectroscopic studies of the self-assembly.

## 2. Results and Discussion

### 2.1. Synthesis and Characterization of Terpyridine Precursors ***1***–***4***

**Scheme 3:**
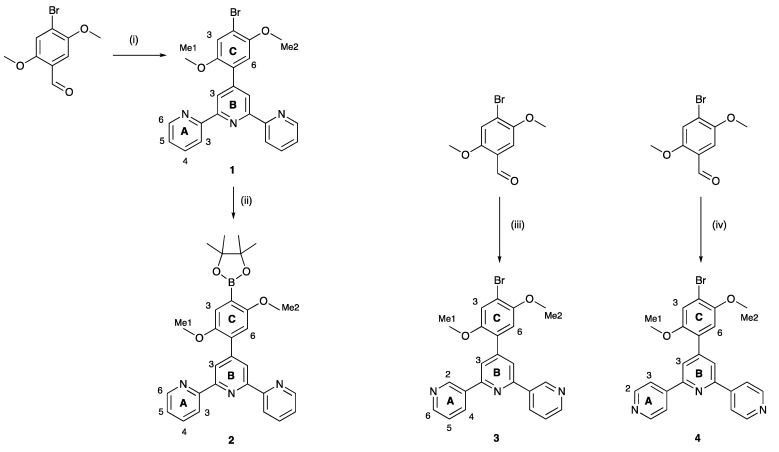
Synthetic routes to compounds **2**–**4**, with labelling for NMR spectroscopic assignments. Reaction conditions: (**i**) 2-acetylpyridine, KOH, NH_3_, EtOH, room temperature (ca. 22 °C), 15 h; (**ii**) B_2_pin_2_, KOAc, [Pd(dppf)Cl_2_] (5 mol%), DMSO, 110 °C, 24 h; (**iii**) 3-acetylpyridine, KOH, NH_3_, EtOH, room temperature (ca. 22 °C), 15 h; (**iv**) 4-acetylpyridine, KOH, NH_3_, EtOH, room temperature (ca. 22 °C), 15 h.

In order to achieve an efficient synthesis of tritopic ligands containing combinations of 2,2′:6′,2″- and 3,2′:6′,3″-tpy or 2,2′:6′,2″- and 4,2′:6′,4″-tpy metal binding domains, we first prepared compound **1** (Scheme 3) using the one-pot method of Wang and Hanan [19]. Bromo-2,5-dimethoxybenzaldehyde was reacted with two equivalents of 2-acetylpyridine in basic EtOH solution followed by the addition of an excess of aqueous NH_3_. Compound **1** was isolated in 43.3% yield. The reaction of **1** with bis(pinacolato)diboron in the presence of [Pd(dppf)Cl_2_] catalyst and KOAc led to **2** (Scheme 3), which was isolated in 50.2% yield. This strategy is analogous to that used by Sun et al. for the preparation of 4′-[4-(4,4,5,5-tetramethyl-1,3,2-dioxaborolan-2-yl)phenyl]-2,2′:6′,2″-terpyridine [20]. Compounds **3** and **4** (Scheme 3) are isomers of **1** and were prepared in an analogous manner using 3- or 4-acetylpyridine in place of 2-acetylpyridine. After recrystallization, **3** and **4** were obtained in 29.2% and 38.0% yields, respectively. The methoxy groups in these compounds were introduced to improve the solubilities of the final ligands **5** and **6** described in Section 2.2.

**Figure 1:**
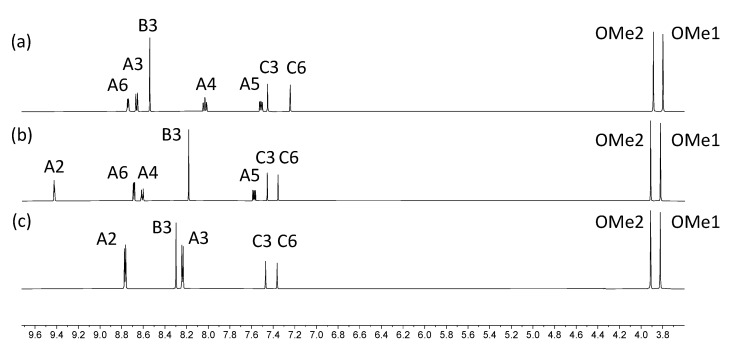
^1^H NMR spectra of compounds (**a**) **1**, (**b**) **3** and (**c**) **4** (500 MHz, DMSO-*d*_6_, 298 K).

**Figure 2:**
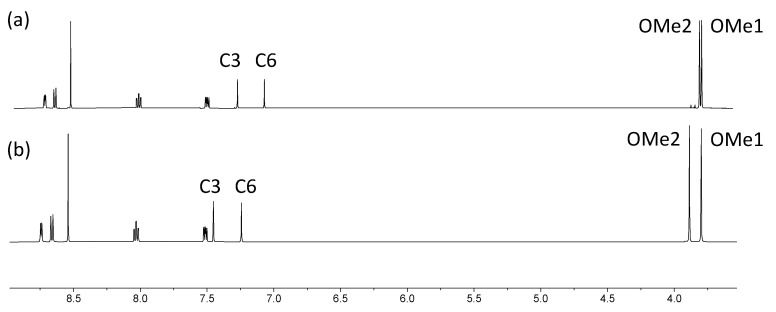
Comparison of the ^1^H NMR spectra of compounds (**a**) **2** and (**b**) **1** (500 MHz, DMSO-d_6_, 298 K). The resonance for the CH_3_ protons in **2** at *δ* 1.32 ppm is not shown (see Figure S11 for the full spectrum).

The electrospray mass spectra of **1–4** were recorded in MeCN solutions with the addition of a few drops of formic acid. The base peak in each spectrum of **1**, **3** and **4** arose from the [M+H]^+^ ion (Figures S1–S3) while for **2**, peaks at *m*/z = 518.21 and 1013.39 (Figure S4) were assigned to the [M+Na]^+^ and [2M+Na]^+^ ions, respectively. The ^1^H NMR spectra of isomers **1**, **3** and **4** are compared in Figure 1, and confirm the characteristic spectroscopic signature of each tpy isomer. The signals arising from the two OMe environments are little affected across the series of compounds. Assignments of the ^1^H and ^13^C{^1^H} NMR spectra were made using COSY, NOESY, HMQC, and HMBC methods (Figures S5–S10). Upon going from **1** to **2**, the aromatic region of the ^1^H NMR spectrum is not significantly affected by the replacement of the bromo substituent by the boron-containing group. The effects of this change are most noticeable for the signals arising from protons H^C6^ and H^C3^, and the OMe groups, as shown in Figure 2. The full ^1^H NMR spectrum, and the HMQC and HMBC spectra for **2** are displayed in Figures S11–S13, and full assignments are given in the Materials and Methods section.

**Figure 3:**
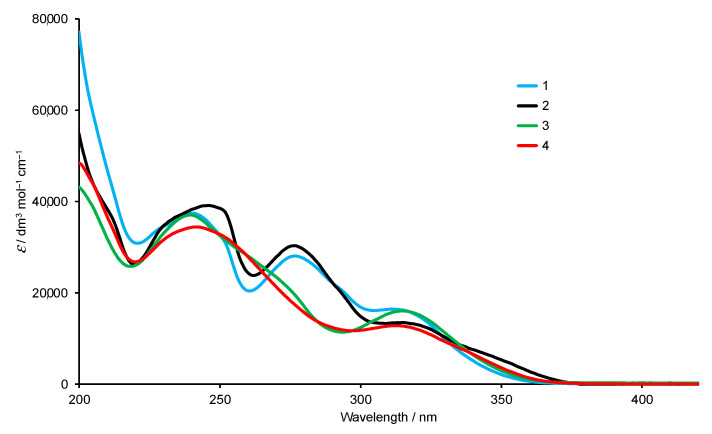
Absorption spectra of compounds **1–4** in MeCN (2.0 × 10^−5^ mol dm^−3^).

The solution absorption spectra of **1–4** (Figure 3) exhibit intense absorptions in the UV region arising mainly from spin-allowed π*←π transitions. Replacing the bromo by the dioxaborolane group has only a small impact on the absorption spectrum. In contrast, moving across the isomer series **1** to **3** to **4** leads to noticeable changes in the profile of the spectrum (Figure 3).

**Scheme 4:**
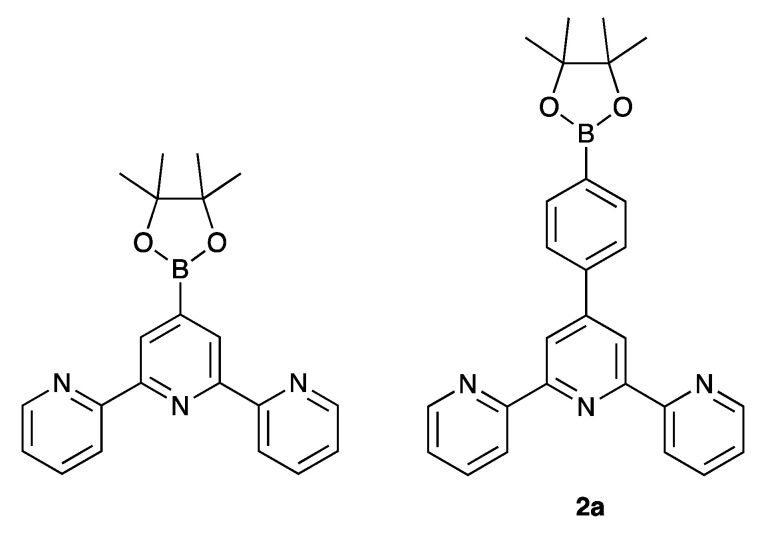
Structures of the previously reported compounds 4′-(4,4,5,5-tetramethyl-1,3,2-dioxaborolan-2-yl)-2,2′:6′,2″-terpyridine [23] and (4,4,5,5-tetramethyl-1,3,2-dioxaborolan-2-yl)phenyl]-2,2′:6′,2″-terpyridine (**2a**) [20].

**Figure 4:**
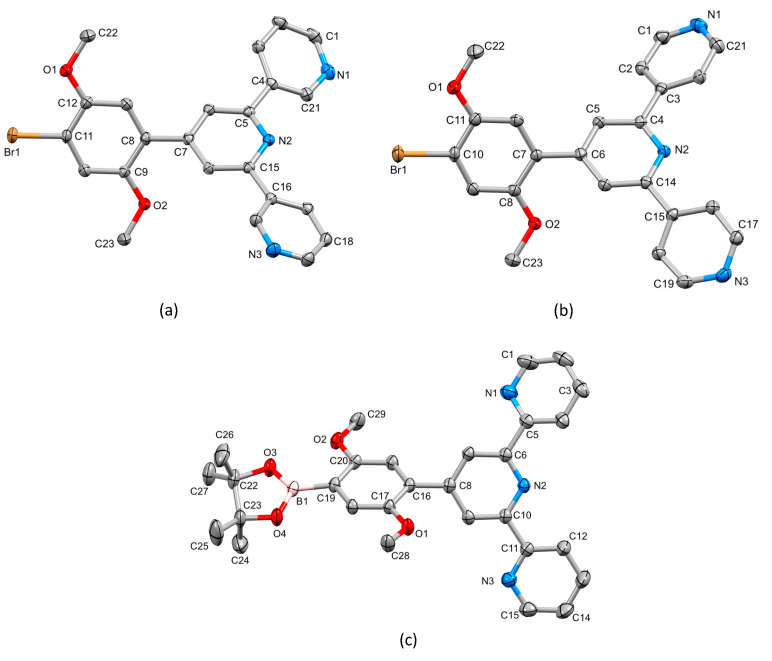
The structures of (**a**) **3**, (**b**) **4** and (**c**) **2** with ellipsoids plotted at 50% probability level and H atoms omitted for clarity. Selected bond parameters: in **3**: C11–Br1 = 1.8950(16) Å, C12–O1 = 1.365(2), O1–C22 = 1.429(2), C9–O2 = 1.367(2), O2–C23 = 1.424(2) Å, C12–O1–C22 = 116.40(14), C9–O2–C23 = 117.14(14)°; in **4**: C10–Br1 = 1.898(2), O1–C11 = 1.358(2), O1–C22 = 1.426(3), O2–C8 = 1.370(2), O2–C23 = 1.437(2) Å, C11–O1–C22 = 117.91(17), C8–O2–C23 = 117.08(16)°; in **2**: C19–B1 = 1.561(2), O3–B1 = 1.361(2), O4–B1 = 1.364(2), O1–C17 = 1.3714(18), O1–C28 = 1.4278(18), O2–C20 = 1.3682(18), O2–C29 = 1.4226(19), O3–C22 = 1.4676(19), O4–C23 = 1.4643(19) Å, C17–O1–C28 = 117.28(12), C20–O2–C29 = 117.62(12), O4–B1–C19 = 119.46(14), O3–B1–O4 = 113.21(14), O3–B1–C19 = 127.33(15)°.

The single crystal structures of **2**, **3** and **4** were obtained. X-ray quality crystals of **2** were obtained directly from the isolated crystalline solid of **2** (see Section 3.3). Single crystals of **3** were grown from an EtOH/CHCl_3_ solution stored at 2–5 °C for three days, and X-ray quality crystals of **4** were selected from the crystalline solid after the isolation of the bulk compound (see Section 3.5). Due to the similarities in their structures, we discuss the compounds together, starting at a molecular level and then moving on to the packing interactions. Compounds **3** and **4** crystallize in the triclinic space group $P\bar{1}$ and monoclinic space group *P*2_1_/*n*, respectively, while **2** crystallizes in the orthorhombic space group *Pbca*. The asymmetric unit in each structure contains one independent molecule, and these are depicted in Figure 4. Bond lengths and angles are unexceptional, and selected parameters are given in the caption to Figure 4. In all three compounds, the O–C_arene_ bonds are shorter than the O–C_Me_ bonds (see caption to Figure 4) and this is consistent with an extension of the p-conjugation from the arene ring to an sp^2^ hybridized O atom. This is also supported by the C_Me_–O–C_arene_ bond angle which lie in the range 116.40(14) to 117.91(17)°. In **2**, the boron atom is planar (sum of the C–B–O and O–B–O bond angles = 360°), and the bond lengths O3–B1 = 1.361(2) Å and O4–B1 = 1.364(2) Å are consistent with a p-contribution to the B–O bonds [21]. The angles between the least squares planes of adjacent pairs of pyridine rings containing N1/N2 and N2/N3 are 28.1 and 25.9° in **3**, 30.3 and 21.6° in **4**, and 26.2 and 9.5° in **2**. In each compound, the arene ring exhibits the typical twist with respect to the pyridine ring containing N2 in order to relieve repulsive H…H contacts (angle = 44.1° in **3**, 43.5° in **4** and 50.3° in **2**). We note that Schwalbe et al. have reported the structure of 4′-(4,4,5,5-tetramethyl-1,3,2-dioxaborolan-2-yl)-2,2′:6′,2″-terpyridine (Scheme 4), and that in this compound, the BO_2_C_2_-ring (although slightly puckered) is approximately coplanar with the central pyridine ring of the tpy unit (Cambridge Structural Database [22], CSD, refcode FEKWEZ) [23]. In 4′-[4-(4,4,5,5-tetramethyl-1,3,2-dioxaborolan-2-yl)phenyl]-2,2′:6′,2″-terpyridine (**2a**, Scheme 4), the angles between the least squares planes of the central pyridine ring and the phenylene spacer, and the phenylene spacer and the BO_2_C_2_-ring (again, slightly puckered) are 23.5 and 3.4°, and 39.6 and 10.6°, respectively, for two independent molecules (CSD refcode NERZAO) [20].

**Scheme 5:**
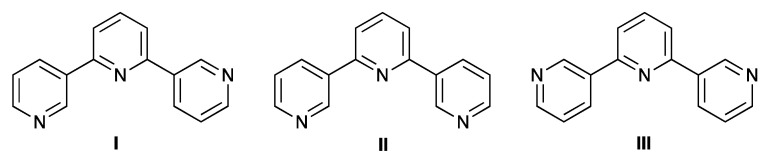
The three limiting planar conformations, **I–III**, of 3,2′:6′,3″-terpyridine. Conformation **I** is observed in the single crystal structure of **3**.

**Figure 5:**
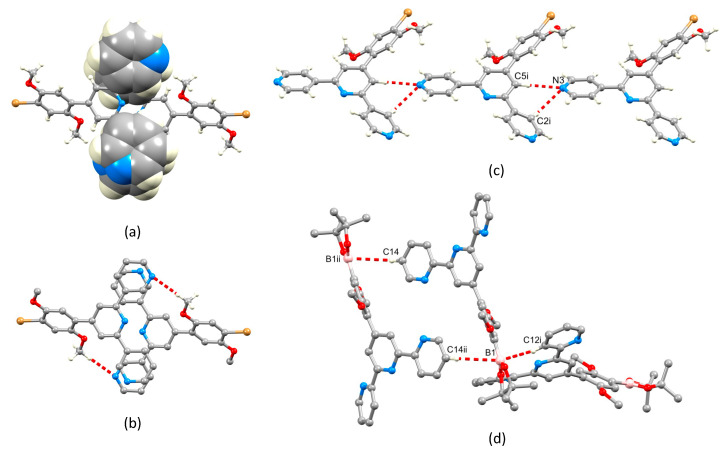
(**a**,**b**) Packing of molecules of **3**: (**a**) face-to-face π-stacking of pyridine rings, and (**b**) C–H…N hydrogen bonds within the same structural motif as that shown in part (**a**). (**c**) Bifurcated hydrogen bonding interactions in **4**; symmetry code i = *x*, *y*, –1 + *z*. (**d**) C–H…B1 contacts in the packing of molecules of **2**; symmetry codes: i = ^3^/_2_–*x*, 1–*y*, ^1^/_2_+*z*; ii = 2–*x*, 1–*y*, 1–*z*.

A *trans,trans*-conformation of the 2,2′:6′,2″-tpy unit is observed in **2** (Figure 4c), typical of non-coordinated tpy ligands. The conformation of the 3,2′:6′,3″-tpy unit in **3** (Figure 4a) is one of three possible, limiting planar conformations (Scheme 5) and appears to be adopted in the crystal structure of **3** because of the assembly of the centrosymmetric motif shown in Figure 5. This features a combination of face-to-face π-stacking of pyridine rings containing N3 and N1^i^ (symmetry code i = 2 − *x*, 1 − *y*, 1 − *z*) and C23–H23…N1^i^ hydrogen bonds (C23 is in one of the methyl groups, see Figure 4a). For the π-stacking interaction (Figure 5a), the centroid…centroid distance is 3.93 Å, and the angle between the ring planes is 29.2°. While these parameters are not optimal [24], the interaction is supplemented by CH_methyl_…N hydrogen bonds (Figure 5b) for which the metric parameters are C23…N1^i^ = 3.518(4) Å, C23H23…N1^i^ = 2.63 Å, and angle C23–H23…N1^i^ = 150.6°. In addition, C3–H3…Br1^ii^ (symmetry code ii = 1 − *x*, 1 − *y*, − *z*) contacts also play a role in packing interactions (C3…Br^ii^ = 3.586(2) Å, C3H3…Br^ii^ = 2.99 Å, angle C3–H3…Br^ii^ = 122.1°). In contrast to the packing interactions in **3**, there are no π-stacking interactions between molecules of **4**, and packing is dominated by CH…N and CH…O hydrogen bonds. Of note are the bifurcated interactions shown in Figure 5c which involve N3 as a single donor and C2^i^–H2^i^ and C5^i^–H5^i^ as acceptors. Pertinent bond metrics are C2^i^…N3 = 3.396(3) and C5^i^…N3 = 3.558(3) Å, C2^i^H2^i^…N3 = 2.74 Å and C5^i^H5^i^…N3 = 2.61 Å, angle C2^i^–H2^i^…N3 = 126.7° and C5^i^–H5^i^…N3 = 175.2°.

Molecules in the crystal structure are oriented with respect to one another such that the trigonal planar B1 atom exhibits short contacts with two C–H units from adjacent molecules (Figure 5d). The contacts involve C12^i^–H12^i^…B1 and C14^ii^–H14^ii^…B1 (metrics are C12^i^…B1 = 3.660(3) and C14^ii^…B1 = 3.889(3) Å; H12^i^…B1 = 2.94 and H14^ii^…B1 = 3.04 Å; angle C12^i^–H12^i^…B1 = 133.8 and C14^ii^–H14^ii^…B1 = 149.4°). Although not discussed in the original work [20], the crystal structure of **2a** (Scheme 4) also exhibits short C–H…B contacts, but in this case, they are augmented by C–H…O contacts. A search of the CSD [22] (version 2022.2.0) using Conquest (version 2022.2.0) [25] for compounds containing a Bpin unit with a trigonal planar boron atom attached to an aryl of alkenyl unit (see Figure S14 in the Supporting Materials for the search motif) gave 487 compounds, 261 of which contained C–H…B contacts in which the H…B distance was less than or equal to the sum of the van der Waals radii; normalized H coordinates were applied within the program Conquest [25] to make the bond length equal to the average neutron diffraction value. Disordered structures were excluded. The range of CH…B distances was 2.58–3.02 Å with a mean value of 3.02 Å, and the C–H…B angles ranged from 100.6 to 177.9° with a mean value of 145.1°. The parameters for the interactions in **2** are comparable to these mean values. The nature of the interaction is ambiguous: the boron atom could act as a Lewis acid with the C–H bond as a donor, or the C–H σ* orbital could accept electron density from the π-bonding orbitals of the B–O or B–C_aryl/alkenyl_ bonds.

### 2.2. Synthesis and Characterization of the Asymmetrical Bis(Terpyridine) Ligands ***5*** and ***6***

**Scheme 6:**
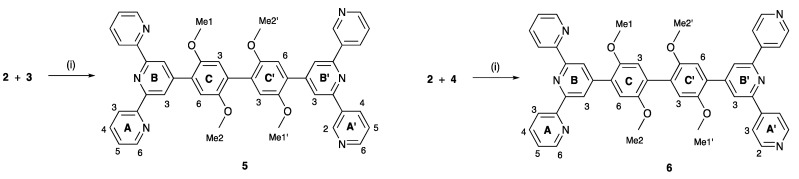
Synthetic routes to compounds **5** and **6** with labelling for NMR spectroscopic assignments. Conditions: (**i**) Na_2_CO_3_, [Pd(dppf)Cl_2_], DMSO, 110 °C, 15 h.

**Figure 6:**
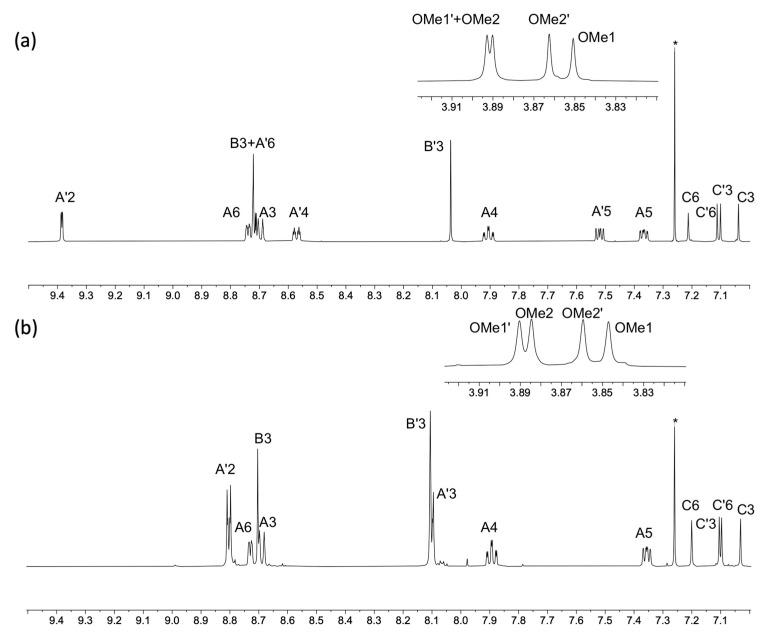
A comparison of the aromatic regions of the ^1^H NMR spectra of compounds (**a**) **5** and (**b**) **6** with the OMe region shown in the insets (500 MHz, CDCl_3_, 298 K). * = residual CHCl_3_. For atom labelling, see Scheme 6. HMQC and HMBC spectra are shown in Figures S17–S20 in the Supporting Materials.

Compounds **5** and **6** were prepared using palladium-catalyzed cross coupling reactions of the boronic ester **2** with the two bromo-derivatives **3** and **4** (Scheme 6). The asymmetric bis(terpyridines) **5** and **6** were isolated in 57.4 and 36.2% yields, respectively, after purification. Both electrospray and high-resolution ESI mass spectra were recorded and showed peaks assigned to [M+H]^+^. For **5**, the base peak in the HR ESI-MS arose from the [M+H]^+^ ion (*m*/*z* 737.2869) and the [M+Na]^+^ ion was also observed at higher mass (Figure S15 in the Supporting Materials). For **6**, peaks at *m*/*z* 369.1476 and 737.2871 arose from the [M+2H]^2+^ and [M+H]^+^ ions, with the former corresponding to the base peak as shown in Figure S16. The assignments of the ^1^H and ^13^C{^1^H} NMR spectra of **5** and **6** were made with the aid of COSY, NOESY, HMQC and HMBC experiments, and by comparing the spectra of **5** and **6** with those of compounds **1**, **3** and **4**. NOESY crosspeaks between H^B3^/H^C6^, H^B′3^/H^C′6^, H^C3^/H^OMe1^, H^C′3^/H^OMe1′^, H^C6^/H^OMe2^, and H^C′6^/H^OMe2′^ (see Scheme 6) were diagnostic. Figure 6 displays a comparison of the ^1^H NMR spectra of **5** and **6**. In addition to the signatures of the 2,2′:6′,2″- and 3,2′:6′,3″-tpy domains in the spectrum of **5**, and of the 2,2′:6′,2″- and 4,2′:6′,4″-tpy domains in that of **6**, both spectra show a clean separation of the signals for the four chemically and magnetically different OMe environments. The absorption spectra of **5** and **6** are discussed in the next section.

### 2.3. Expanded Ligands [Fe(***5***)_2_]^2+^ and [Fe(***6***)_2_]^2+^

The expanded ligands [Fe(**5**)_2_]^2+^ and [Fe(**6**)_2_]^2+^ were isolated as the nitrate and tetrafluoridoborate salts, respectively. The salt [Fe(**5**)_2_][NO_3_]_2_ was prepared from the corresponding chloride. Ligand **5** was dissolved in a mixture of MeOH and CHCl_3_, and addition of FeCl_2_·4H_2_O followed by an excess of aqueous NaNO_3_ (see Section 3.8) led to the precipitation of [Fe(**5**)_2_][NO_3_]_2_ as a purple solid in 82.4% yield. Compound **6** was reacted with Fe(BF_4_)_2_·6H_2_O in a mixture of MeOH and CHCl_3_ (see Section 3.9), and [Fe(**6**)_2_][BF_4_]_2_ was isolated as a purple solid in 78.0% yield.

**Figure 7:**
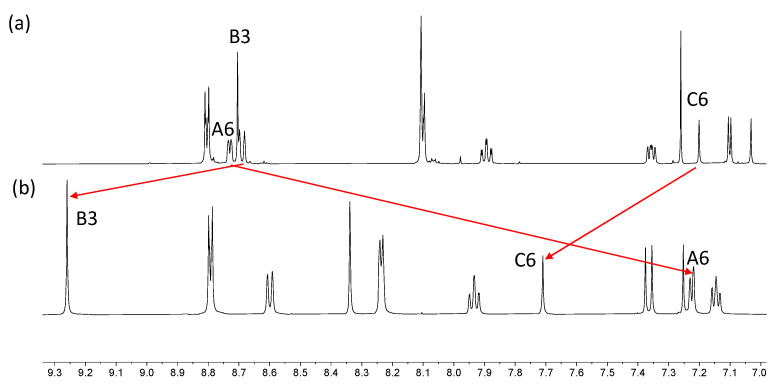
Comparison of the aromatic regions of the ^1^H NMR spectra (500 MHz, 298 K) for (**a**) compound **6** in CDCl_3_, and (**b**) [Fe(**6**)_2_][BF_4_]_2_ in CD_3_CN. Differences in solubilities required the use of different solvents.

The electrospray and HR-ESI mass spectra of both iron(II) complexes were recorded, and each spectrum exhibited a peak arising from the [M–2BF_4_]^2+^ ion (*m*/*z* 764.67). The characteristic isotope pattern and half-mass peak separations are depicted in Figures S21 and S22. In the HR-ESI mass spectrum of [Fe(**6**)_2_][BF_4_]_2_, a low intensity peak was observed at *m*/*z* 1615.4979 corresponding to the [M–BF_4_]^+^ ion. The ^1^H and ^13^C{^1^H} NMR spectra were recorded in CD_3_CN; the addition of a few grains of solid K_2_CO_3_ to the solutions in the NMR tubes led to a sharpening of the signals. The spectra were assigned using NOESY, COSY, HMQC, and HMBC methods and through comparison with the corresponding uncoordinated **5** and **6**. Figures S23–S26 show the HMQC and HMBC. Coordination of ligands **5** and **6** to iron(II) results in significant changes in the chemical shifts of the signals for protons H^A6^, H^B3^ and H^C6^ (Figure 7 and Figure S27). These protons are all associated with the 2,2′:6′,2″-tpy metal-binding domain (see Scheme 6). The large shift to lower frequency for H^A6^ is typical of the formation of {M(2,2′:6′,2″-tpy)_2_}*^n^*^+^ units as the H^A6^ protons of one ligand experience shielding since these protons lie over the aromatic system of the second ligand. Protons H^B3^ and H^C6^ are both influenced by the change in conformation of the 2,2′:6′,2″-tpy from *trans,trans* to *cis,cis* upon coordination to Fe(II).

**Figure 8:**
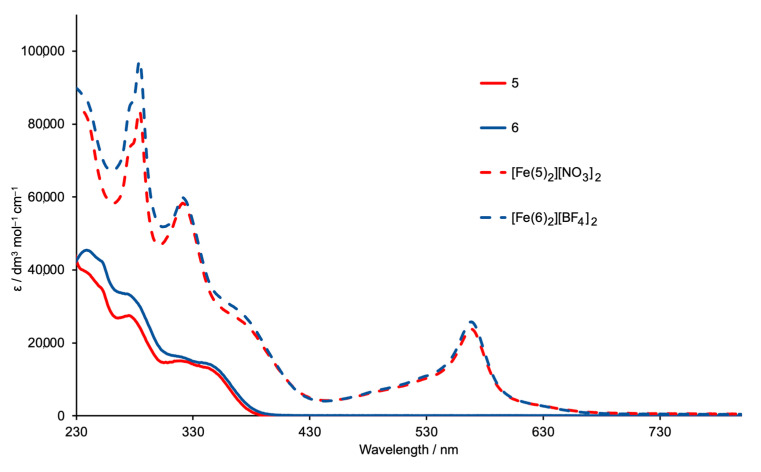
Absorption spectra of ligands **5** (MeCN/CHCl_3_, see text) and **6** (MeCN, 2.0 × 10^−5^ mol dm^−3^), and of the complexes [Fe(**5**)_2_][NO_3_]_2_ and [Fe(**6**)_2_][BF_4_]_2_ (MeCN, 1.0 × 10^−5^ mol dm^−3^).

Figure 8 shows the solution absorption spectra of the two bis(terpyridines) **5** and **6** and their iron(II) complexes. For solubility reasons, the absorption spectrum of **5** was recorded in a MeCN/CHCl_3_. The stock solution (1.0 × 10^−3^ mol dm^−3^) for the measurements was made up in a mixture of MeCN/CHCl_3_ (9:1 by volume) which was then diluted to a concentration of 2.0 × 10^−5^ mol dm^−3^ using MeCN. MeCN solutions were used for the other three compounds. The spectra of **5** and **6** exhibit intense absorptions in the UV region arising mainly from spin-allowed π*←π transitions. The approximate doubling of the extinction coefficients in the high-energy region on going from free ligand to complex is consistent with the formation of [FeL_2_]^2+^ species. The spectra of [Fe(**5**)_2_][NO_3_]_2_ and [Fe(**6**)_2_][BF_4_]_2_ exhibit broad absorptions with *λ*_max_ = 568 nm which are assigned to metal-to-ligand charge transfer (MLCT).

**Figure 9:**
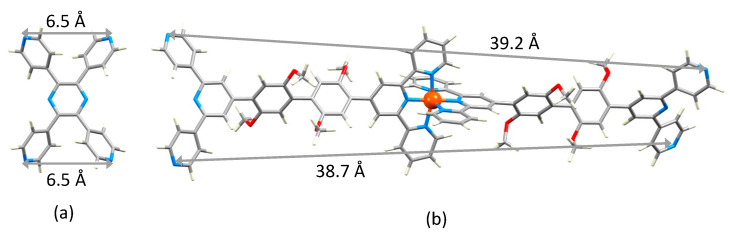
Modelled structures of (**a**) tetra(pyridin-4-yl)pyrazine and (**b**) [Fe(**6**)_2_]^2+^.

Attempts to grow X-ray quality crystals of [Fe(**5**)_2_][NO_3_]_2_ and [Fe(**6**)_2_][BF_4_]_2_ were unsuccessful. Attempts were also made using [PF_6_]^−^ in place of [NO_3_]^−^ in the case of [Fe(**5**)_2_]^2+^, and with mixtures of the two counterions. In the absence of a single crystal structure, the structure of the [Fe(**6**)_2_]^2+^ ion was optimized at a molecular mechanics level (MM2) using the program Spartan 18 (v. 1.4.8) [26], and the dimensions of this “expanded ligand” were compared with a related ‘simple ligand’. The simplest analogue is tetra(pyridin-4-yl)pyrazine. While structures of two zinc(II) complexes of this ligand have been reported [27], the structure of the ligand itself has not. The modelled structure (MM2 level [26]) is shown in Figure 9a and the distance between the two 4,2′:6′,4″-tpy donor sites of 6.5 Å is compared to the separation of the corresponding units in [Fe(**6**)_2_]^2+^ (Figure 9b). Comparisons with other bis(4,2′:6′,4″-tpy) ligands could also be made, but we have chosen only the smallest analog in keeping with the comparisons made in Scheme 1. In the case of the [Fe(**5**)_2_]^2+^ ion, the conformational variation of the 3,2′:6′,3″-tpy units compared to that of the 4,2′:6′,4″-tpy domains leads to a more flexible expanded ligand.

## 3. Materials and Methods

### 3.1. General

^1^H, ^13^C{^1^H} and 2D NMR spectra were recorded at 298 K on a Bruker Avance iii-500 spectrometer (Bruker BioSpin AG, Fällanden, Switzerland) equipped with a BBFO probehead. The ^1^H and ^13^C NMR chemical shifts were referenced with respect to residual solvent peaks (*δ* TMS = 0). A Shimadzu LCMS-2020 instrument (Shimadzu Schweiz GmbH, 4153 Reinach, Switzerland) was used to record electrospray ionization (ESI) mass spectra, and a Bruker maXis 4G QTOF (Bruker BioSpin AG, Fällanden, Switzerland) instrument for HR ESI mass spectra. A PerkinElmer UATR Two instrument (PerkinElmer, 8603 Schwerzenbach, Switzerland) and a Shimadzu UV2600 (Shimadzu Schweiz GmbH, 4153 Reinach, Switzerland) spectrophotometer were used to record FT-IR and absorption spectra, respectively.

Bis(pinacolato)diboron, 4-bromo-2,5-dimethoxybenzaldehyde, 2-acetylpyridine, 3-acetylpyridine, and [Pd(dppf)Cl_2_] were purchased from Fluorochem (Chemie Brunschwig AG, 4052 Basel, Switzerland), 4-acetylpyridine and Fe(BF_4_)_2_·6H_2_O from Sigma Aldrich (Sigma Aldrich Chemie GmbH, 89,555 Steinheim, Germany), and Na_2_CO_3_ and FeCl_2_·4H_2_O from Acros Organics (Fisher Scientific AG, 4153 Reinach, Switzerland). These chemicals were used as received.

### 3.2. Compound ***1***

The compound 4-bromo-2,5-dimethoxybenzaldehyde (5.00 g, 20.4 mmol) was dissolved in EtOH (60 mL), then 2-acetylpyridine (4.94 g, 4.57 mL, 40.8 mmol) and crushed KOH (2.29 g, 40.8 mmol) were added to the solution. Aqueous NH_3_ (32%, 78.6 mL) was slowly added to the reaction mixture, and this was stirred at room temperature overnight. The solid that formed was collected by filtration, washed with H_2_O (3 × 10 mL) and EtOH (3 × 10 mL), recrystallized from EtOH/CHCl_3_ and dried in vacuo. Compound **1** was isolated as a white powder (3.96 g, 8.84 mmol, 43.3%). M.p. = 175 °C. ^1^H NMR (500 MHz, DMSO-*d*_6_) *δ*/ppm 8.74 (ddd, *J* = 4.8, 1.8, 0.9 Hz, 2H, H^A6^), 8.66 (dt, *J* = 8.0, 1.1 Hz, 2H, H^A3^), 8.54 (s, 2H, H^B3^), 8.03 (td, 2H, *J* = 7.6, 1.8 Hz, H^A4^), 7.51 (ddd, 2H, *J* = 7.6, 4.8, 1.1 Hz, H^A5^), 7.45 (s, 1H, H^C3^), 7.24 (s, 1H, H^C6^), 3.89 (s, 3H, H^OMe2^), 3.80 (s, 3H, H^OMe1^). ^13^C{^1^H} NMR (126 MHz, DMSO-*d*_6_) *δ*/ppm 155.1 (C^B2/A2^), 154.9 (C^B2/A2^), 150.5 (C^C2/C5^), 150.0 (C^C2/C5^), 149.4 (C^A6^), 147.3 (C^B4^), 137.4 (C^A4^), 127.5 (C^C1^), 124.4 (C^A5^), 121.0 (C^A3/B3^), 120.9 (C^A3/B3^), 117.2 (C^C3^), 114.2 (C^C6^), 111.7 (C^C4^), 56.9 (C^OMe1/OMe2^), 56.7 (C^OMe1/OMe2^). UV-VIS (MeCN, 2.0 × 10^−5^ mol dm^−3^) *λ*/nm (*ε*/dm^3^ mol^−1^ cm^−1^): 240 (37,500), 277 (28,100), 311 *sh* (16,500). ESI-MS *m*/*z* 448.02 [M+H]^+^ (calc. 448.06). Found C 61.04, H 3.99, N 9.42; required for C_23_H_18_BrN_3_O_2_ C 61.62, H 4.05, N 9.37.

### 3.3. Compound ***2***

A 100 mL Schlenk tube was charged with compound **1** (1.00 g, 2.23 mmol), B_2_pin_2_ (0.680 g, 2.68 mmol), KOAc (0.657 g, 6.69 mmol) and [Pd(dppf)Cl_2_] (0.049 g, 0.067 mmol). The reaction vessel was flushed with nitrogen, then degassed DMSO (25 mL) was added, and the mixture was stirred and heated at 110 °C for 24 h. After allowing it to cool to room temperature, the mixture was diluted with toluene (100 mL) and was washed with brine (4 × 50 mL). The toluene layer was dried over Na_2_SO_4_ and was then filtered. The solvent was removed by rotary evaporation, yielding a brown residue, which was redissolved in CH_2_Cl_2_ and filtered through a celite pad. The brown portion was retained by the celite, while the colorless solution was dried, giving **2** (0.555 g, 1.12 mmol, 50.2%) as a crystalline, white solid. M.p. = 211 °C. ^1^H NMR (500 MHz, DMSO-*d*_6_) *δ*/ppm 8.75 (ddd, *J* = 4.8, 1.8, 0.9 Hz, 2H, H^A6^), 8.67 (dt, *J* = 8.0, 1.1 Hz, 2H, H^A3^), 8.55 (s, 2H, H^B3^), 8.04 (td, 2H, *J* = 7.6, 1.8 Hz, H^A4^), 7.52 (ddd, 2H, *J* = 7.6, 4.8, 1.1 Hz, H^A5^), 7.29 (s, 1H, H^C3^), 7.08 (s, 1H, H^C6^), 3.78 (s, 3H, H^OMe2^), 3.77 (s, 3H, H^OMe1^), 1.32 (s, 12H, H^OMe3^). ^13^C{^1^H} NMR (126 MHz, DMSO-*d*_6_) *δ*/ppm 158.3 (C^C5^), 155.1 (C^B2^), 154.8 (C^A2^), 149.7 (C^C2^), 149.4 (C^A6+B4^), 147.9 (C^C4^), 137.5 (C^A4^), 131.1 (C^C1^), 124.4 (C^A5^), 121.0 (C^B3/A3^), 120.9 (C^B3/A3^), 119.3 (C^C3^), 113.2 (C^C6^), 83.4 (C^a^), 56.3 (C^OMe1+OMe2^), 24.7 (C^OMe3^). UV-VIS (MeCN, 2.0 × 10^−5^ mol dm^−3^) *λ*/nm (*ε*/dm^3^ mol^−1^ cm^−1^): 246 (39,200), 276 (30,300), 315 sh (13,500). ESI-MS *m*/*z* 518.21 [M+Na]^+^ (calc. 518.22), 1013.40 [2M+Na]^+^ (calc. 1013.46). Found C 69.66, H 6.07, N 8.55; required for C_29_H_30_BN_3_O_4_ C 70.31, H 6.10, N 8.48.

### 3.4. Compound ***3***

The compound 4-bromo-2,5-dimethoxybenzaldehyde was dissolved in EtOH (40 mL), then 3-acetylpyridine (2.47 g, 2.25 mL, 20.4 mmol) and crushed KOH (1.15 g, 20.4 mmol) were added to the solution. Aqueous NH_3_ (32%, 39.3 mL) was added slowly to the reaction mixture, and this was stirred at room temperature overnight. The solid that formed was collected by filtration, washed with H_2_O (3 × 10 mL) and EtOH (3 × 10 mL), recrystallized from EtOH/CHCl_3_ and dried under vacuum. Compound **3** was isolated as a white crystalline solid (1.33 g, 2.98 mmol, 29.2%). M.p. = 198 °C. ^1^H NMR (500 MHz, DMSO-*d*_6_) *δ*/ppm 9.43 (dd, *J* = 2.4, 0.9 Hz, 2H, H^A2^), 8.69 (dd, *J* = 4.8, 1.6 Hz, 2H, H^A6^), 8.61 (ddd, *J* = 8.0, 2.4, 1.6 Hz, 2H, H^A4^), 8.19 (s, 2H, H^B3^), 7.58 (ddd, *J* = 8.0, 4.8, 0.9 Hz, 2H, H^A5^), 7.46 (s, 1H, H^C3^), 7.36 (s, 1H, H^C6^), 3.92 (s, 3H, H^OMe2^), 3.83 (s, 3H, H^OMe1^). ^13^C{^1^H} NMR (126 MHz, DMSO-*d*_6_) *δ*/ppm 154.0 (C^B2^), 150.6 (C^C2^), 150.1 (C^C5^), 149.9 (C^A6^), 148.2 (C^A2^), 147.6 (C^B4^), 134.4 (C^A4^), 134.0 (C^A3^), 127.0 (C^C1^), 123.9 (C^A5^), 120.4 (C^B3^), 117.1 (C^C3^), 114.9 (C^C6^), 111.8 (C^C4^), 56.9 (C^OMe2^), 56.7 (C^OMe1^). UV-VIS (MeCN, 2.0 × 10^−5^ mol dm^−3^) *λ*/nm (*ε*/dm^3^ mol^−1^ cm^−1^): 240 (37,000), 315 (16,000). ESI-MS *m*/*z* 448.03 [M+H]^+^ (calc. 448.06). Found C 61.18, H 4.03, N 9.42; required for C_23_H_18_BrN_3_O_2_ C 61.62, H 4.05, N 9.37.

### 3.5. Compound ***4***

The compound 4-bromo-2,5-dimethoxybenzaldehyde (2.50 g, 10.2 mmol) was dissolved in EtOH (40 mL), then 4-acetylpyridine (2.47 g, 2.27 mL, 20.4 mmol) and crushed KOH (1.15 g, 20.4 mmol) were added to the solution. Aqueous NH_3_ (32%, 39.3 mL) was slowly added to the reaction mixture, and this was stirred at room temperature overnight. The crystalline solid that formed was collected by filtration, washed with H_2_O (3 × 10 mL) and EtOH (3 × 10 mL), and dried in vacuo. Compound **4** was isolated as a white crystalline solid (1.74 g, 3.87 mmol, 38.0%). M.p. = 242 °C. ^1^H NMR (500 MHz, DMSO-*d*_6_) *δ*/ppm 8.77 (m, 4H, H^A2^), 8.30 (s, 2H, H^B3^), 8.24 (m, 4H, H^A3^), 7.47 (s, 1H, H^C3^), 7.36 (s, 1H, H^C6^), 3.91 (s, 3H, H^OMe2^), 3.82 (s, 3H, H^OMe1^). ^13^C{^1^H} NMR (126 MHz, DMSO-*d*_6_) *δ*/ppm 153.7 (C^B2^), 150.6 (C^C2^), 150.4 (C^A2^), 150.0 (C^C5^), 147.9 (C^B4^), 145.3 (C^A4^), 126.7 (C^C1^), 121.8 (C^B3^), 121.1 (C^A3^), 117.1 (C^C3^), 114.9 (C^C6^), 112.0 (C^C4^), 56.9 (C^OMe2^), 56.7 (C^OMe1^). UV-VIS (MeCN, 2.0 × 10^−5^ mol dm^−3^) *λ*/nm (*ε*/dm^3^ mol^−1^ cm^−1^): 242 (34,400), 313 (12,800). ESI-MS *m*/*z* 448.03 [M+H]^+^ (calc. 448.06). Found C 60.93, H 3.99, N 9.36; required for C_23_H_18_BrN_3_O_2_ C 61.62, H 4.05, N 9.37.

### 3.6. Compound ***5***

A 100 mL Schlenk tube was charged with **2** (500 mg, 1.01 mmol), **3** (498 mg, 1.11 mmol), Na_2_CO_3_ (321 mg, 3.03 mmol) and [Pd(dppf)Cl_2_] (37.0 mg, 0.05 mmol). The reaction vessel was flushed with N_2_, and then degassed DMSO (20 mL) was added, and the mixture was stirred at 110 °C under an N_2_ atmosphere overnight (ca. 14 h). The reaction mixture was then cooled to room temperature, leading to some precipitation. The solid was removed by filtration and was washed with toluene. The filtrate was diluted with toluene (70 mL) and was washed with brine (5 × 100 mL), then the organic layer was dried over Na_2_SO_4_, filtered, and the solvent was partially removed by rotary evaporation. While removing the solvent, part of the product precipitated as an off-white solid. This was collected by filtration and washed with acetone, yielding a first batch of **5**. The filtrate was then further concentrated and purified by column chromatography on basic Al_2_O_3_ with Brockmann activity II (ethyl acetate:cyclohexane 1:2). After combining batches of product, **5** was isolated as a white solid (428 mg, 0.58 mmol, 57.4%). M.p. = 294 °C. ^1^H NMR (500 MHz, CDCl_3_) *δ*/ppm 9.39 (dd, *J* = 2.3, 0.9 Hz, 2H, H^A′2^), 8.74 (ddd, *J* = 4.8, 1.8, 0.9 Hz, 2H, H^A6^), 8.73–8.71 (m, 4H, H^A′6+B3^), 8.70 (dt, *J* = 8.0, 1.1 Hz, 2H, H^A3^), 8.57 (ddd, *J* = 8.0, 2.3, 1.7 Hz, 2H, H^A′4^), 8.04 (s, 2H, H^B′3^), 7.91 (td, 2H, *J* = 7.7, 1.8 Hz, H^A4^), 7.52 (ddd, 2H, *J* = 8.0, 4.8, 0.8 Hz, H^A′5^), 7.37 (ddd, 2H, *J* = 7.6, 4.8, 1.2 Hz, H^A5^), 7.21 (s, 1H, H^C6^), 7.11 (s, 1H, H^C′6^), 7.10 (s, 1H, H^C′3^), 7.04 (s, 1H, H^C3^), 3.89 (m, 6H, H^OMe1′+OMe2^), 3.86 (s, 3H, H^OMe2′^), 3.85 (s, 3H, H^OMe1^). ^13^C{^1^H} NMR (126 MHz, CDCl_3_) *δ*/ppm 156.5 (C^B2/A2^), 155.4 (C^B2/A2^), 154.7 (C^B′2^), 151.7 (C^C′5^), 151.4 (C^C2^), 150.8 (C^C5^), 150.6 (C^C′2^), 149.7 (C^A′6^), 149.2 (C^A6^), 148.9 (C^C′4^), 148.6 (C^C4^), 148.2 (C^A′2^), 137.1 (C^A4^), 135.3 (C^A′3)^, 135.2 (C^A′4^), 129.7 (C^C4^), 128.7 (C^C1^), 128.1 (C^C′4^), 127.3 (C^C′1^), 124.0 (C^A′5^), 123.9 (C^A5^), 122.0 (C^B3^), 121.6 (C^A3^), 120.6 (C^B′3^), 115.6 (C^C′3^), 115.4 (C^C3^), 114.4 (C^C6^), 114.3 (C^C′6^), 57.05 (C^OMe1/OMe1′/OMe2/OMe2′^), 57.0 (C^OMe1/OMe1′/OMe2/OMe2′^), 56.7 (C^OMe1/OMe1′/OMe2/OMe2′^), 56.6 (C^OMe1/OMe1′/OMe2/OMe2′^). UV-VIS (MeCN/CHCl_3_ 9:1, 2.0 × 10^−5^ mol dm^−3^) *λ*/nm (*ε*/dm^3^ mol^−1^ cm^−1^) 275 (28,900), 318 (15,600). ESI-MS (MeCN solution with addition of a few drops of formic acid) *m*/*z* 737.23 [M+H]^+^ (calc. 737.29). HR ESI-MS *m*/*z* 737.2869 [M+H]^+^ (base peak, calc. 737.2871), 759.2687 [M+Na]^+^ (calc. 759.2690).

### 3.7. Compound ***6***

A 100 mL Schlenk tube was charged with **2** (750 mg, 1.67 mmol), **4** (753 mg, 1.52 mmol), Na_2_CO_3_ (483 mg, 4.56 mmol) and [Pd(dppf)Cl_2_] (55.6 mg, 0.076 mmol). The reaction vessel was flushed with N_2_, then degassed DMSO (20 mL) was added, and the mixture was stirred at 110 °C under N_2_ overnight (ca. 14 h). The reaction mixture was then cooled to room temperature, leading to some precipitation. The solid was removed by filtration and washed with toluene. The filtrate was diluted with additional toluene (70 mL) and washed with brine (5 × 100 mL), then the organic layer was dried over Na_2_SO_4_, filtered, and the solvent was partially removed by rotary evaporation. A first batch of **6** (174 mg) was obtained by washing the brown residue with acetone and recrystallizing it from acetone/chloroform. The rest of the compound was purified by column chromatography on basic Al_2_O_3_ with Brockmann activity II (ethyl acetate:cyclohexane 2:1). After combining batches of product, **6** was isolated as a white solid (407 mg, 0.55 mmol, 36.2%). M.p. = 227 °C. ^1^H NMR (500 MHz, CDCl_3_): *δ*/ppm 8.80 (d, *J* = 6.12 Hz, 4H, H^A′2^), 8.73 (ddd, *J* = 4.7, 1.9, 0.9 Hz, 2H, H^A6^), 8.71–8.67 (m, 4H, H^A3+B3^), 8.12–8.09 (m, 6H, H^A′3+B′3^), 7.89 (td, 2H, *J* = 7.7, 1.8 Hz, H^A4^), 7.36 (ddd, 2H, *J* = 7.5, 4.7, 1.2 Hz, H^A5^), 7.20 (s, 1H, H^C6^), 7.11 (s, 1H, H^C′3^), 7.10 (s, 1H, H^C′6^), 7.03 (s, 1H, H^C3^), 3.89 (s, 3H, H^OMe1′^), 3.88 (s, 3H, H^OMe2^), 3.86 (s, 3H, H^OMe2′^), 3.85 (s, 3H, H^OMe1^). ^13^C{^1^H} NMR (126 MHz, CDCl_3_): *δ*/ppm 156.6 (C^B2^), 155.5 (C^A2^), 154.7 (C^B′2^), 151.7 (C^C′5^), 151.3 (C^C2^), 150.8 (C^C5^), 150.6 (C^C′2^), 150.4 (C^A′2^), 149.3 (C^A6^), 149.1 (C^B′4^), 148.5 (C^B4^), 146.7 (C^A′4^), 137.0 (C^A4^), 129.8 (C^C′4^), 128.8 (C^C1^), 128.0 (C^C4^), 127.1 (C^C′1^), 123.9 (C^A5^), 121.9 (C^B3^), 121.8 (C^A′3/B′3/A3^), 121.6 (C^A′3/B′3/A3^), 121.5 (C^A′3/B′3/A3^), 115.6 (C^C′3^), 115.3 (C^C3^), 114.4 (C^C6^), 114.3 (C^C′6^), 57.1 (C^OMe1/OMe2/OMe1′/OMe2′^), 57.0 (C^OMe1/OMe2/OMe1′/OMe2′^), 56.7 (C^OMe1/OMe2/OMe1′/OMe2′^), 56.6 (C^OMe1/OMe2/OMe1′/OMe2′^). UV-VIS (MeCN, 2.0 × 10^−5^ mol dm^−3^) *λ*/nm (*ε*/dm^3^ mol^−1^ cm^−1^): 238 (44,500), 254 sh (40,500), 275 sh (32,900), 343 sh (14,000). ESI-MS (MeCN solution with addition of a few drops of formic acid) *m*/*z* 759.28 [M+Na]^+^ (base peak, calc. 759.27), 737.31 [M+H]^+^ (calc. 737.29). HR ESI-MS *m*/*z* 369.1476 [M+2H]^2+^ (base peak, calc. 369.1472), 737.2869 [M+H]^+^ (calc. 737.2871).

### 3.8. [Fe(***5***)_2_][NO_3_]_2_

An MeOH solution of FeCl_2_·4H_2_O (3.37 mg, 0.017 mmol) was added dropwise to a warm solution (70 °C) of **5** (25 mg, 0.034 mmol) in MeOH/CHCl_3_ (40 mL, 1:3 by vol.). The mixture turned immediately purple while stirring. After 30 min, CHCl_3_ was removed under vacuum following which the purple MeOH solution was treated with an excess of aqueous NaNO_3_, and the mixture was stirred for an additional 10 min; precipitation of a purple solid was observed. The mixture was cooled to 2–5 °C in a refrigerator. When the solution became colorless and all the fine purple solid had precipitated, the solid was collected on celite, washed with H_2_O, and dissolved in MeCN. The solvent was removed by rotary evaporation, affording [Fe(**5**)_2_][NO_3_]_2_ as a purple solid (23.2 mg, 0.014 mmol, 82.4%). ^1^H NMR (500 MHz, CD_3_CN) *δ*/ppm 9.46 (dd, *J* = 2.3, 0.9 Hz, 4H, H^A′2^), 9.27 (s, 4H, H^B3^), 8.70 (dd, *J* = 4.7, 1.6 Hz, 4H, H^A′6^), 8.64 (dt, *J* = 7.9, 1.9 Hz, 4H, H^A′4^), 8.61 (m, 4H, H^A3^), 8.24 (s, 4H, H^B′3^), 7.93 (td, *J* = 7.7, 1.5 Hz, 4H, H^A4^), 7.72 (s, 2H, H^C6^), 7.55 (ddd, *J* = 8.1, 4.8, 0.9 Hz, 4H, H^A′5^), 7.38 (s, 2H, H^C′6^), 7.35 (s, 2H, H^C3^), 7.24 (s, 2H, H^C′3^), 7.23 (dd, *J* = 6.2, 1.2 Hz, 4H, H^A6^), 7.14 (ddd, *J* = 7.1, 5.6, 1.3 Hz, 4H, H^A5^), 4.10 (s, 6H, H^OMe1^), 4.03 (s, 6H, H^OMe2^), 3.95 (s, 6H, H^OMe1′^), 3.93 (s, 6H, H^OMe2′^). ^13^C{^1^H} NMR (126 MHz, CD_3_CN): *δ*/ppm 160.7 (C^B2^), 159.2 (C^A2^), 155.6 (C^B′2^), 154.0 (C^A6^), 152.8 (C^C5^), 152.5 (C^C′5^), 151.9 (C^C2^), 151.5 (C^C′2^), 151.1 (C^A′6^), 149.4 (C^A′2^), 149.1 (C^B4^), 139.7 (C^A4^), 135.6 (C^A′3^), 135.4 (C^A′4^), 131.7 (C^C4^), 129.7 (C^C′4^), 128.4 (C^C′1^), 128.2 (C^A5^), 126.3 (C^C1^), 125.1 (C^B3^), 124.7 (C^A′5^), 124.6 (C^A3^), 121.5 (C^B′3^), 116.8 (C^C3^), 116.5 (C^C′3^), 115.3 (C^C6^), 115.1 (C^C′6^), 57.4 (C^OMe2+OMe1^), 57.2 (C^OMe2′+OMe1′^). UV-VIS (MeCN, 1.0 × 10^−5^ mol dm^−3^) *λ*/nm (*ε*/dm^3^ mol^−1^ cm^−1^): 284 (83,600), 321 (58,300), 568 (23,700). ESI-MS *m*/*z* 764.67 [M–2NO_3_]^2+^ (calc. 764.25). HR-MS *m*/*z* 764.2479 [M–2NO_3_]^2+^ (calc. 764.2469).

### 3.9. [Fe(***6***)_2_][BF_4_]_2_

An MeOH solution of Fe(BF_4_)_2_·6H_2_O (23.0 mg, 0.068 mmol) was added dropwise to a hot solution (70 °C) of **6** (100.0 mg, 0.136 mmol) in MeOH/CHCl_3_ (ca. 1:3). The mixture immediately turned purple while stirring. After 1 h, an excess of aqueous NaBF_4_ was added and the mixture was stirred for an additional 10 min; immediate precipitation of a purple solid was observed. The mixture was cooled down at 2–5 °C. When the solution became colorless and all the fine solids had precipitated, the latter was collected on celite, washed with H_2_O, and dissolved in MeCN. The solvent was removed by rotary evaporation, affording [Fe(**6**)_2_][BF_4_]_2_ as a purple solid (90.3 mg, 0.053 mmol, 78.0%). ^1^H NMR (500 MHz, CD_3_CN with a small amount of added K_2_CO_3_) *δ*/ppm 9.26 (s, 4H, H^B3^), 8.79 (d, *J* = 6.0 Hz, 8H, H^A′2^), 8.60 (d, *J* = 8.0 Hz, 4H, H^A3^), 8.34 (s, 4H, H^B′3^), 8.24 (dd, 8H, H^A′3^), 7.93 (td, 4H, *J* = 7.7, 1.5 Hz, H^A4^), 7.71 (s, 2H, H^C6^), 7.38 (s, 2H, H^C′6^), 7.35 (s, 2H, H^C3^), 7.25 (s, 2H, H^C′3^), 7.22 (m, 4H, H^A6^), 7.15 (ddd, *J* = 7.1, 5.6, 1.3 Hz, 4H, H^A5^), 4.11 (s, 6H, H^OMe1^), 4.03 (s, 6H, H^OMe2^), 3.95 (s, 6H, H^OMe1′^), 3.93 (s, 6H, H^OMe2′^). ^13^C{^1^H} NMR (126 MHz, CD_3_CN, with a small amount of added K_2_CO_3_) *δ*/ppm 160.7 (C^B2^), 159.2 (C^A2^), 155.4 (C^B′2^), 154.0 (C^A6^), 152.8 (C^C5^), 152.6 (C^C′5^), 151.9 (C^C2^), 151.5 (C^C′2^), 151.4 (C^A′2^), 149.7 (C^B′4^), 149.0 (C^B4^), 146.9 (C^A′4^), 139.7 (C^A4^), 131.6 (C^C4^), 129.9 (C^C′4+C′1^), 128.2 (C^A5^), 126.3 (C^C1^), 125.1 (C^B3^), 124.7 (C^A3^), 122.8 (C^B′3^), 122.2 (C^A′3^), 116.8 (C^C3^), 116.5 (C^C′3^), 115.3 (C^C6^), 115.0 (C^C′6^), 57.4 (C^OMe2+OMe1^), 57.2 (C^OMe2′+OMe1′^). UV-VIS (MeCN, 1.0 × 10^−5^ mol dm^−3^) *λ*/nm (*ε*/dm^3^ mol^−1^ cm^−1^): 284 (97,200), 321 (59,800), 568 (25,700). ESI-MS *m*/*z* 764.67 [M–2BF_4_]^2+^ (calc. 764.25). HR-MS *m*/*z* 764.2466 [M–2BF_4_]^2+^ (calc. 764.2469), 1615.4979 [M–BF_4_]^+^ (calc. 1615.4970).

### 3.10. Crystallography

Single crystal data were collected on a Bruker APEX-II CCD diffractometer (CuK*α* radiation) with data reduction, solution, and refinement using the programs APEX [28], ShelXT [29], Olex2 [30], and ShelXL v. 2014/7 [31]. All H atoms were included at geometrically calculated positions and refined using a riding model with *U*_iso_ = 1.2 of the parent atom, with the exception of methyl groups, which were refined with *U*_iso_ = 1.5. Structure analysis and structural diagrams used CSD Mercury 2022.1.0 [32].

### 3.11. Compound ***2***

C_29_H_30_BN_3_O_4_, *M*_r_ = 495.37, colorless block, orthorhombic, space group *Pbca*, *a* = 15.2294(7), *b* = 17.9085(7*)*, *c* = 19.3318(8) Å, *V* = 5272.5(4) Å^3^, *D*_c_ = 1.248 g cm^−3^, *T* = 150 K, *Z* = 8, μ(CuK*_α_*) = 0.668 mm^−1^. Total 65,360 reflections, 4886 unique (*R*_int_ = 0.0460). Refinement of 4044 reflections (340 parameters) with *I* > 2σ(*I*) converged at final *R*_1_ = 0.0414 (*R*_1_ all data = 0.0527), *wR*_2_ = 0.1006 (*wR*_2_ all data = 0.1074), gof = 1.040. CCDC 2203621.

### 3.12. Compound ***3***

C_23_H_18_BrN_3_O_2_, *M*_r_ = 448.31, colorless block, triclinic, space group $P\bar{1}$, *a* = 8.4228(7), *b* = 11.1275(9), *c* = 11.1387(9) Å, *α =* 69.054(3), *β =* 86.557(3), *γ =* 81.212(4)°*, V* = 963.52(14) Å^3^, *D*_c_ = 1.545 g cm^−3^, *T* = 150 K, *Z* = 2, μ(CuK*_α_*) = 3.115 mm^−1^. Total 10,442 reflections, 3414 unique (*R*_int_ = 0.0205). Refinement of 3377 reflections (264 parameters) with *I* > 2σ*(I*) converged at final *R*_1_ = 0.0252 (*R*_1_ all data = 0.0254), *wR*_2_ = 0.0677 (*wR*_2_ all data = 0.0679), gof = 1.051. CCDC 2203620.

### 3.13. Compound ***4***

C_23_H_18_BrN_3_O_2_, *M*_r_ = 448.31, colorless block, monoclinic, space group *P*2_1_/*n*, *a* = 8.3527(5), *b* = 23.5829(14), *c* = 10.5854(7) Å, *β* = 112.567(3)°*, V* = 1925.5(2) Å^3^, *D*_c_ = 1.547 g cm^−3^, *T* = 150 K, *Z* = 4, μ(CuK*_α_*) = 3.117 mm^−1^. Total 12,341 reflections, 3462 unique (*R*_int_ = 0.0231). Refinement of 3210 reflections (264 parameters) with *I* > 2σ*(I*) converged at final *R*_1_ = 0.0321 (*R*_1_ all data = 0.0347), *wR*_2_ = 0.0817 (*wR*_2_ all data = 0.0840), gof = 1.053. CCDC 2203619.

## 4. Conclusions

We have prepared two tritopic ligands, **5** and **6,** which possess 2,2′:6′,2″- and 3,2′:6′,3″-tpy or 2,2′:6′,2″- and 4,2′:6′,4″-tpy metal-binding units, respectively. The synthetic strategy involved the [Pd(dppf)Cl_2_]-catalyzed coupling of the boronic ester **2** with the bromo-derivatives **3** and **4**. Precursors **1**–**4** were fully characterized, including the single crystal structures of compounds **2**, **3,** and **4**. Treatment of **5** and **6** with iron(II) salts led to the isolation of [Fe(**5**)_2_][NO_3_]_2_ and [Fe(**6**)_2_][BF_4_]_2_, which were characterized by spectroscopic methods; in the solution absorption spectra, the MLCT band for each complex exhibits a value of *λ*_max_ = 568 nm. The complexes [Fe(**5**)_2_]^2+^ and [Fe(**6**)_2_]^2+^ are tetratopic ligands bearing either two 3,2′:6′,3″- or 4,2′:6′,4″-tpy units; they represent expanded versions of tetra(pyridin-4-yl)pyrazine. The coordination chemistry of these new expanded ligands remains to be explored, with the ease of synthesis of [Fe(**5**)_2_]^2+^ and [Fe(**6**)_2_]^2+^ rendering them potential building blocks in 2D- and 3D-coordination networks. There are only a few previous literature examples of tetratopic metallocomplexes with an {Fe(2,2′:6′,2″tpy)_2_} core [33,34].

## Data Availability

After publication, raw data files will be available open access at zenodo.com.

## Supplementary Materials

The following supporting information can be downloaded at: https://www.mdpi.com/article/10.3390/molecules28010082/s1, Figures S1–S4: Electrospray mass spectra of **1–4**; Figures S5–S13: NMR spectra of **1**–**4**; Figure S14: Definition of the Conquest search; Figures S15 and S16: electrospray mass spectra of **5** and **6**; Figures S17–S20: NMR spectra of **5** and **6**; Figures S21 and S22: electrospray mass spectra of [Fe(**5**)_2_][NO_3_]_2_ and [Fe(**6**)_2_][BF_4_]_2_; Figures S23–S26: NMR spectra of [Fe(**5**)_2_][NO_3_]_2_ and [Fe(**6**)_2_][BF_4_]_2_; Figure S27: comparison of the ^1^H NMR spectra of **5** and [Fe(**5**)_2_][NO_3_]_2_.

## Sample Availability

No samples of compounds are available from the authors.

## References

[1] Damhus T., Hartshorn R.M., Hutton A.T., Connelly N.G., Damhus T., Hartshorn R.M., Hutton A.T. (2005). Nomenclature of Inorganic Chemistry, IUPAC Recommendations 2005, IUPAC Red Book.

[2] Constable E.C. (2018). A Journey from Solution Self-Assembly to Designed Interfacial Assembly. Adv. Inorg. Chem..

[3] Constable E.C., Housecroft C.E. (2017). More hydra than Janus—Non-classical coordination modes in complexes of oligopyridine ligands. Coord. Chem. Rev..

[4] Gelmini L., Stephan D.W. (1986). The facile preparation of early transition metal/late transition metal heterobimetallic complexes; (η^5^-C_5_H_5_)_2_Zr(PPh_2_)_2_ as a ‘metalloligand’ for Ni, Pd and Pt. Inorg. Chim. Acta.

[5] Kumar G., Kumar G., Gupta R. (2021). Effect of pyridyl donors from organic ligands versus metalloligands on material design. Inorg. Chem. Front..

[6] Li F., Lindoy L.F. (2019). Metalloligand Strategies for Assembling Heteronuclear Nanocages—Recent Developments. Aust. J. Chem..

[7] Dzhardimalieva G.I., Uflyand I.E. (2017). Design and synthesis of coordination polymers with chelated units and their application in nanomaterials science. RSC Adv..

[8] Kumar G., Gupta R. (2013). Molecularly designed architectures—The metalloligand way. Chem. Soc. Rev..

[9] Chen B., Xiang S., Qian G. (2010). Metal—Organic Frameworks with Functional Pores for Recognition of Small Molecules. Acc. Chem. Res..

[10] Constable E.C. (2008). Expanded ligands—An assembly principle for supramolecular chemistry. Coord. Chem. Rev..

[11] Constable E.C., Schofield E. (1998). Metal-directed assembly of a box-like structure. Chem. Commun..

[12] Constable E.C., Dunphy E.L., Housecroft C.E., Kylberg W., Neuburger M., Schaffner S., Schofield E.R., Smith C.B. (2006). Structural development of free or coordinated 4′-(4-pyridyl)-2,2′:6′,2″-terpyridine ligands through N-alkylation: New strategies for metallomacrocycle formation. Chem. Eur. J..

[13] Beves J.E., Constable E.C., Housecroft C.E., Kepert C.J., Price D.J. (2007). The first example of a coordination polymer from the expanded 4,4′-bipyridine ligand [Ru(pytpy)_2_]^2+^ (pytpy = 4′-(4-pyridyl)-2,2′:6′,2″-terpyridine). CrystEngCommun.

[14] Beves J.E., Constable E.C., Housecroft C.E., Neuburger M., Schaffner S. (2007). A palladium (II) complex of 4′-(4-pyridyl)-2,2′:6′,2″-terpyridine: Lattice control through an interplay of stacking and hydrogen bonding effects. Inorg. Chem. Commun..

[15] Beves J.E., Constable E.C., Housecroft C.E., Kepert C.J., Price D.J., Schaffner S. (2007). The conjugate acid of bis{4′-(4-pyridyl)-2,2′:6′,2″-terpyridine}iron(II) as a self-complementary hydrogen-bonded building block. CrystEngCommun.

[16] Constable E.C., Housecroft C.E., Neuburger M., Schaffner S., Schaper F. (2006). Preparation and structural characterisation of bis(4′-(3-pyridyl)-2,2′:6′,2″-terpyridine)ruthenium(II) hexafluorophosphate. Inorg. Chem. Commun..

[17] Constable E.C., Housecroft C.E., Neuburger M., Schaffner S., Schaper F. (2006). The solid-state structure of bis(4′-(4-pyridyl)-2,2′:6′,2″-terpyridine)ruthenium hexafluorophosphate nitrate—an expanded 4,4′-bipyridine. Inorg. Chem. Commun..

[18] Constable E.C., Dunphy E.L., Housecroft C.E., Neuburger M., Schaffner S., Schaper F., Batten S.R. (2007). Expanded ligands: Bis(2,2′:6′,2″-terpyridine carboxylic acid)ruthenium(II) complexes as metallosupramolecular analogues of dicarboxylic acids. Dalton Trans..

[19] Wang J., Hanan G.S. (2005). A Facile Route to Sterically Hindered and Non-hindered 4′-Aryl-2,2′:6′,2″-terpyridines. Synlett.

[20] Sun Q., Tang L., Zhang Z., Zhang K., Xie Z., Chi Z., Zhang H., Yang W. (2018). Bright NUV mechanofluorescence from a terpyridine-based pure organic crystal. Chem. Commun..

[21] Housecroft C.E., Sharpe A.G. (2018). Inorganic Chemistry.

[22] Groom C.R., Bruno I.J., Lightfoot M.P., Ward S.C. (2016). The Cambridge Structural Database. Acta Cryst..

[23] Schwalbe M., Metzinger R., Teets T.S., Nocera D.G. (2012). Terpyridine–Porphyrin Hetero-Pacman Compounds. Chem. Eur. J..

[24] Janiak C. (2000). A critical account on π–π stacking in metal complexes with aromatic nitrogen-containing ligands. J. Chem. Soc. Dalton Trans..

[25] Bruno I.J., Cole J.C., Edgington P.R., Kessler M., Macrae C.F., McCabe P., Pearson J., Taylor R. (2002). New software for searching the Cambridge Structural Database and visualising crystal structures. Acta Cryst..

[26] (2020). Spartan.

[27] Chen Y., Yang T., Huang J., Yong H.-Y. (2020). Two Zn(II)-based coordination polymers: Treatment effect on the cardiac arrest induced by anesthesia by regulating Sirt1 expression. Inorg. Nano-Metal Chem..

[28] (2013). Software for the Integration of CCD Detector System Bruker Analytical X-ray Systems.

[29] Sheldrick G.M. (2015). ShelXT-Integrated space-group and crystal-structure determination. Acta Cryst..

[30] Dolomanov O.V., Bourhis L.J., Gildea R.J., Howard J.A.K., Puschmann H. (2009). Olex2: A Complete Structure Solution, Refinement and Analysis Program. J. Appl. Cryst..

[31] Sheldrick G.M. (2015). Crystal Structure Refinement with ShelXL. Acta Cryst..

[32] Macrae C.F., Sovago I., Cottrell S.J., Galek P.T.A., McCabe P., Pidcock E., Platings M., Shields G.P., Stevens J.S., Towler M. (2020). Mercury 4.0: From visualization to analysis, design and prediction. J. Appl. Cryst..

[33] Yang J., Clegg J.K., Jiang Q., Lui X., Yan H., Zhong W., Beves J.E. (2013). Multi-pyridine decorated Fe(ii) and Ru(ii) complexes by Pd(0)-catalysed cross couplings: New building blocks for metallosupramolecular assemblies. Dalton Trans..

[34] Liu S.-L., Chen Q.-W., Zhang Z.-W., Chen Q., Wei L.-Q., Lin N. (2022). Efficient heterogeneous catalyst of Fe(II)-based coordination complexes for Friedel-Crafts alkylation reaction. J. Solid State Chem..

