# Supplementary material for: Expanded Ligands Based upon Iron(II) Coordination Compounds of Asymmetrical Bis(terpyridine) Domains
Source: Molecules. 2022 Dec 22;28(1):82. doi: 10.3390/molecules28010082 (PMC9822043; doi:10.3390/molecules28010082)
Supplement: Supplementary file 1 [file molecules-28-00082-s001.zip › molecules-2106413-supplementary.pdf]

Supporting Material

Expanded Ligands Based upon Iron(II) Coordination Compounds of Asymmetrical bis(terpyridine) Domains

Dalila Rocco, Alessandro Prescimone, Catherine E. Housecroft* and Edwin C. Constable

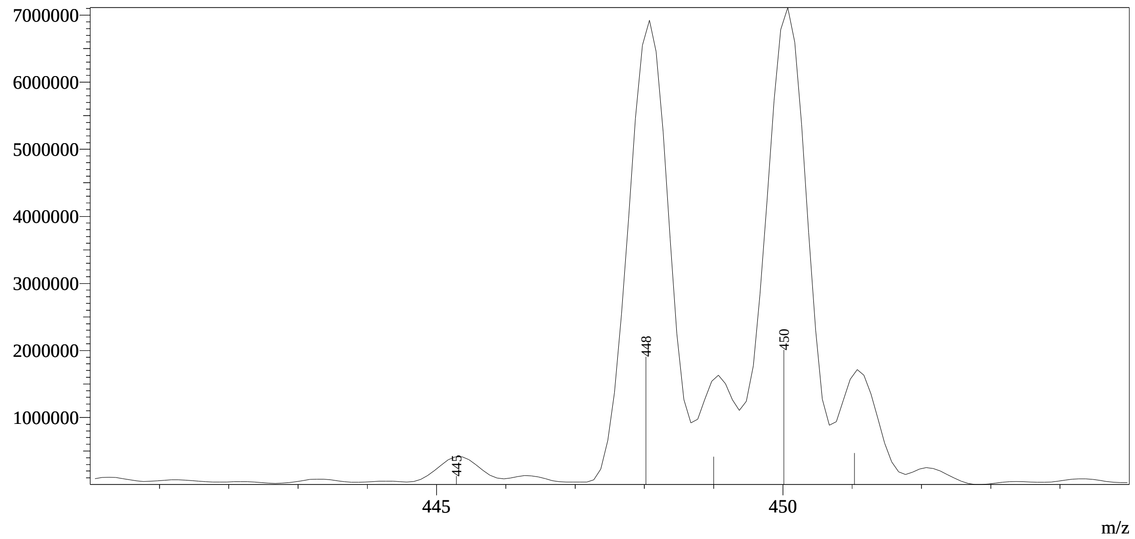

Figure S1. Base peak in the electrospray mass spectrum of **1**.

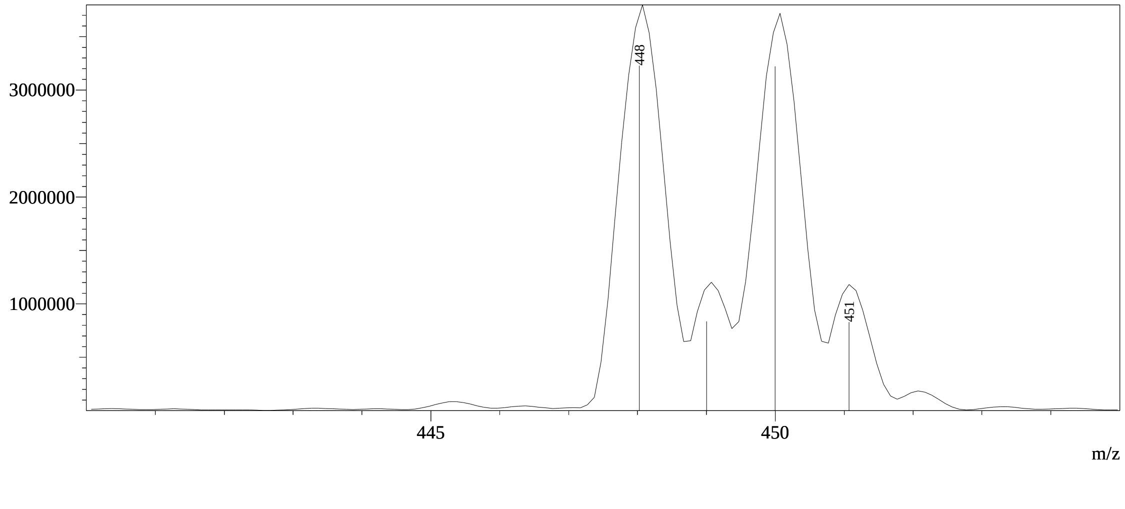

Figure S2. Base peak in the electrospray mass spectrum of **3**.

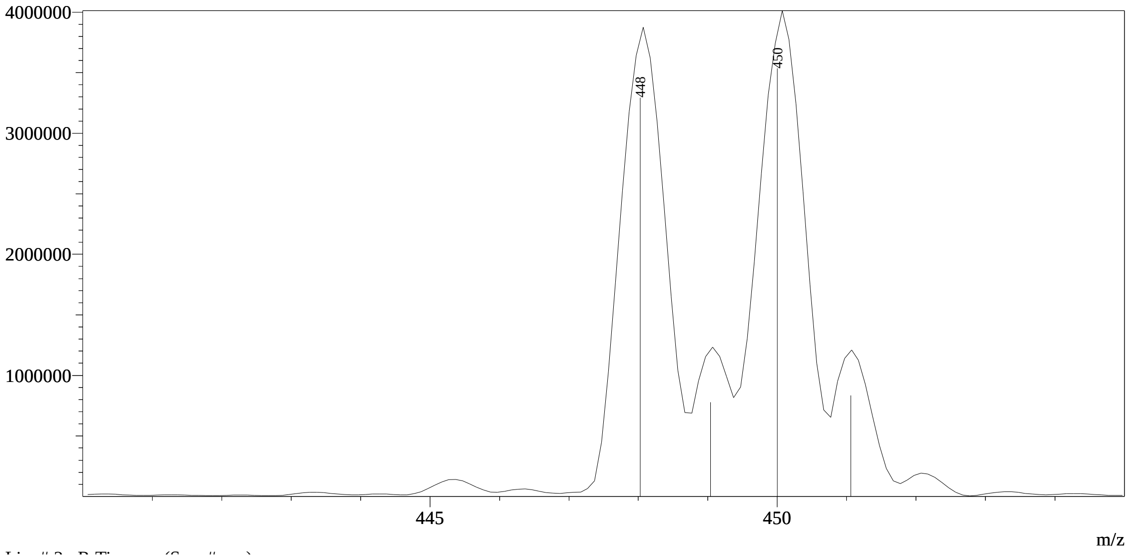

Figure S3. Base peak in the electrospray mass spectrum of **4**.

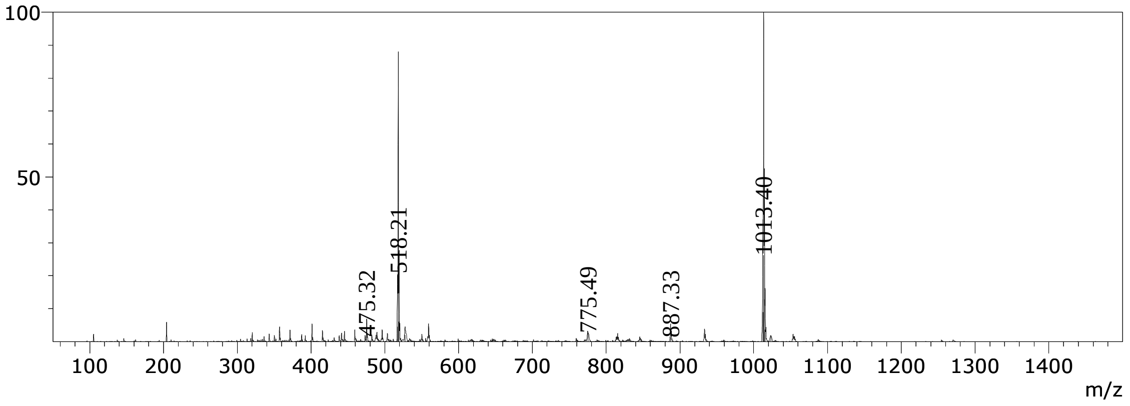

Figure S4. The electrospray mass spectrum of **2**.

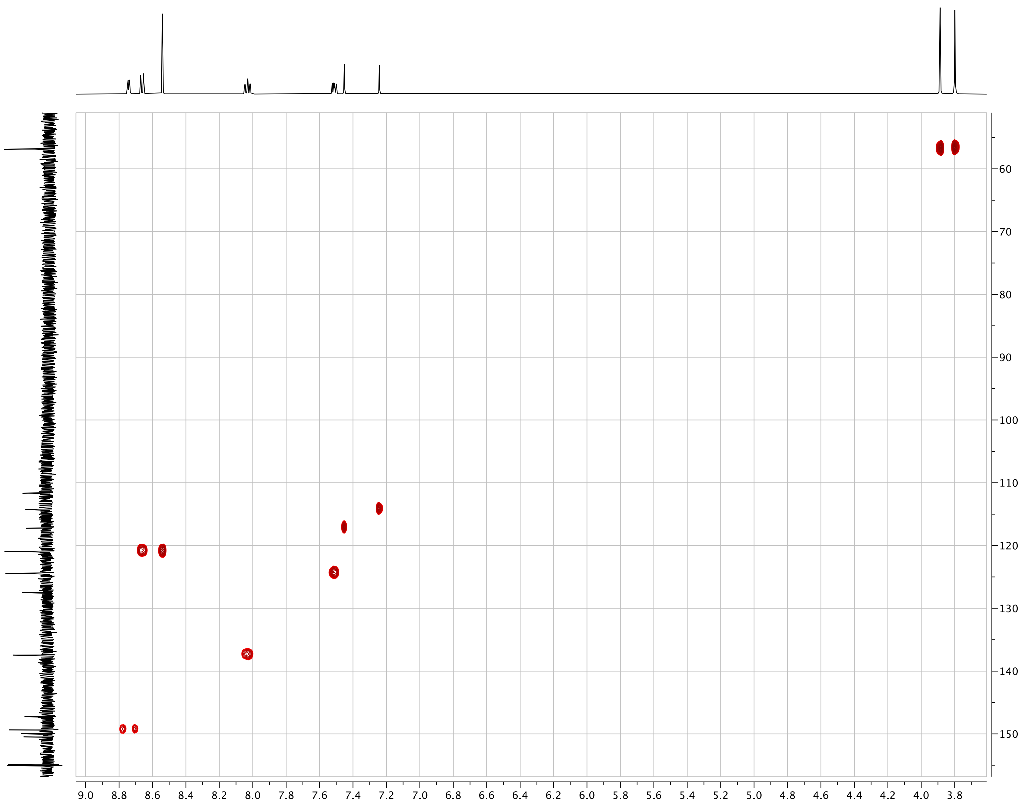

Figure S5. HMQC spectrum of **1** (^{1}H 500 MHz, ^{13}C 126 MHz, DMSO-d_6, 298 K).

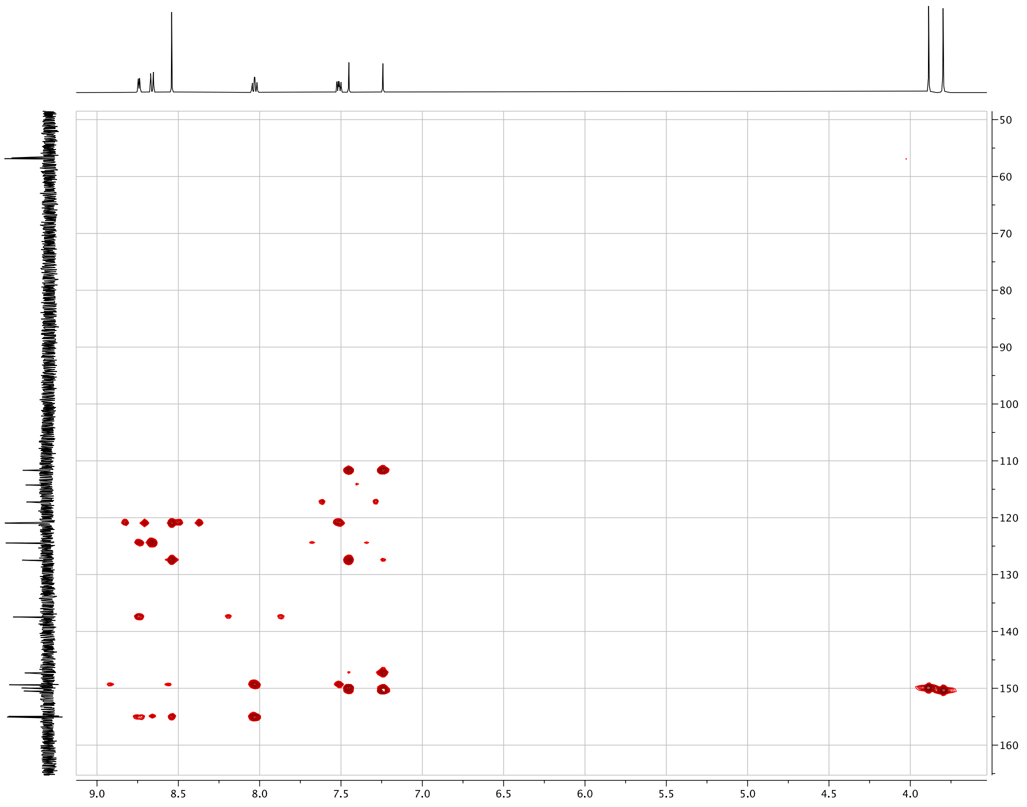

Figure S6. HMBC spectrum of **1** (^{1}H 500 MHz, ^{13}C 126 MHz, DMSO-d_6, 298 K).

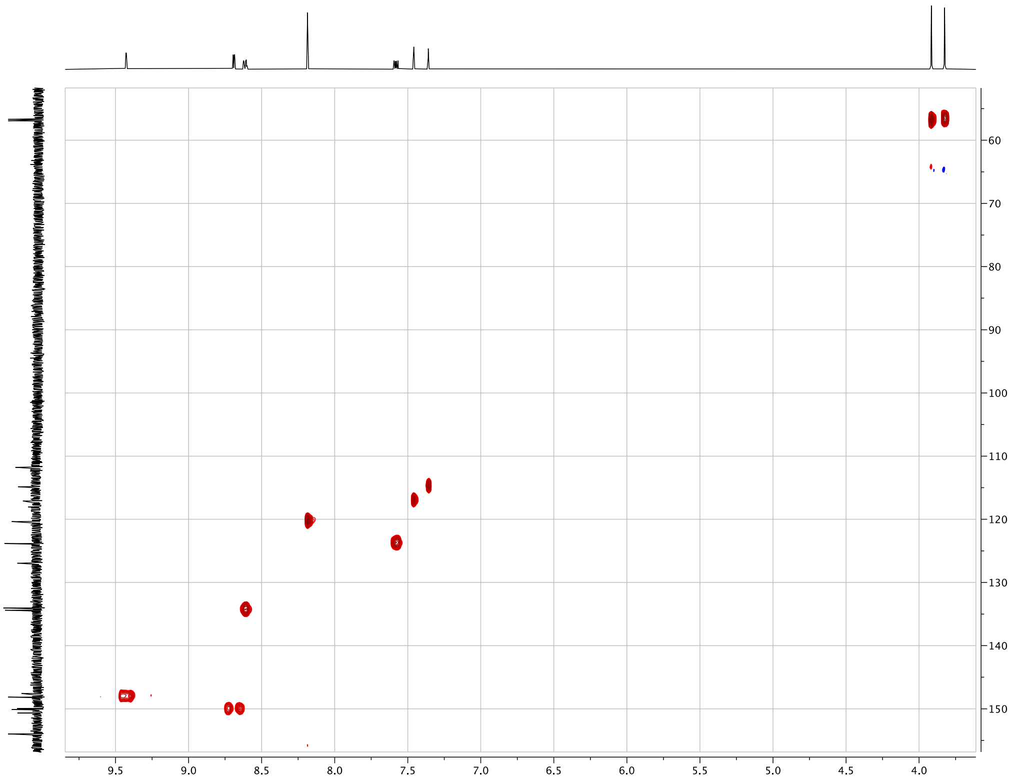

Figure S7. HMQC spectrum of **3** (^{1}H 500 MHz, ^{13}C 126 MHz, DMSO-d_6, 298 K).

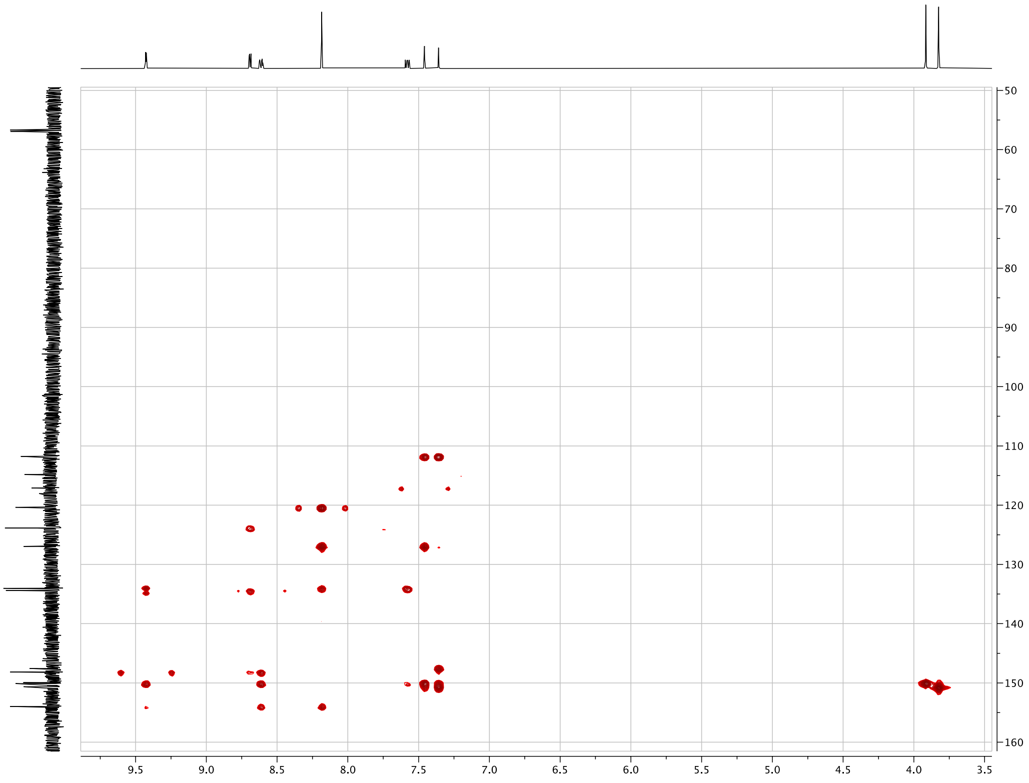

Figure S8. HMBC spectrum of **3** (^{1}H 500 MHz, ^{13}C 126 MHz, DMSO-d_6, 298 K).

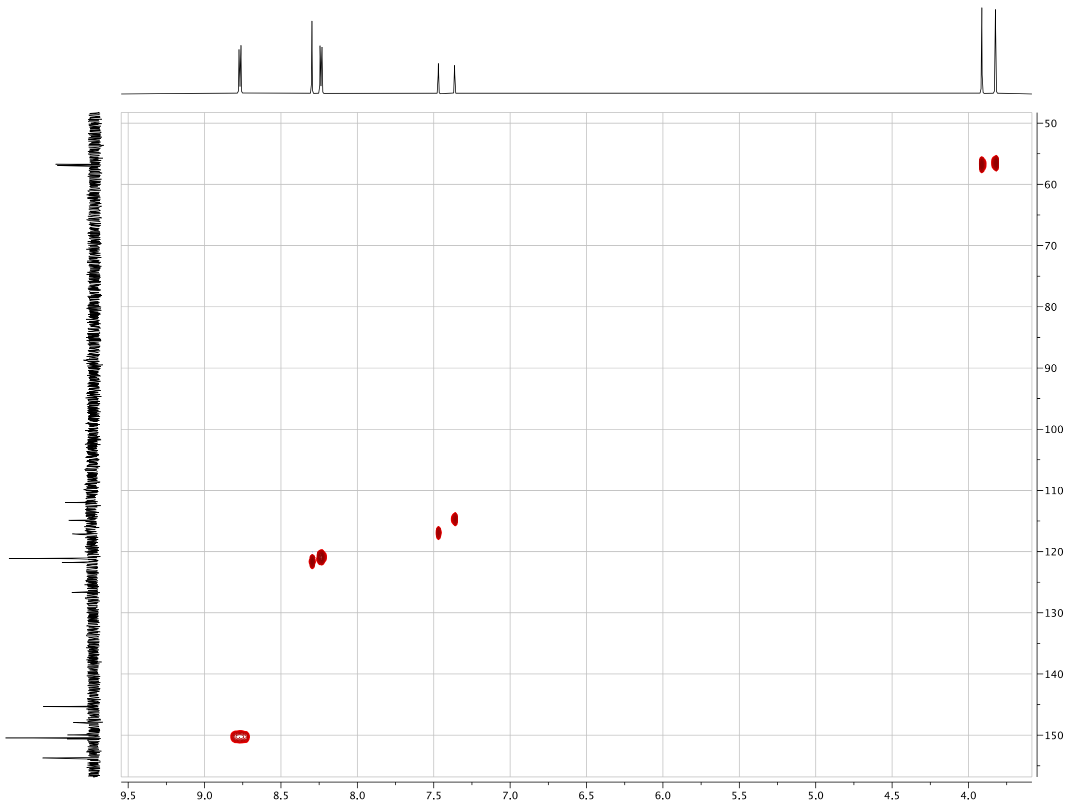

Figure S9. HMQC spectrum of **4** (^{1}H 500 MHz, ^{13}C 126 MHz, DMSO-d_6, 298 K).

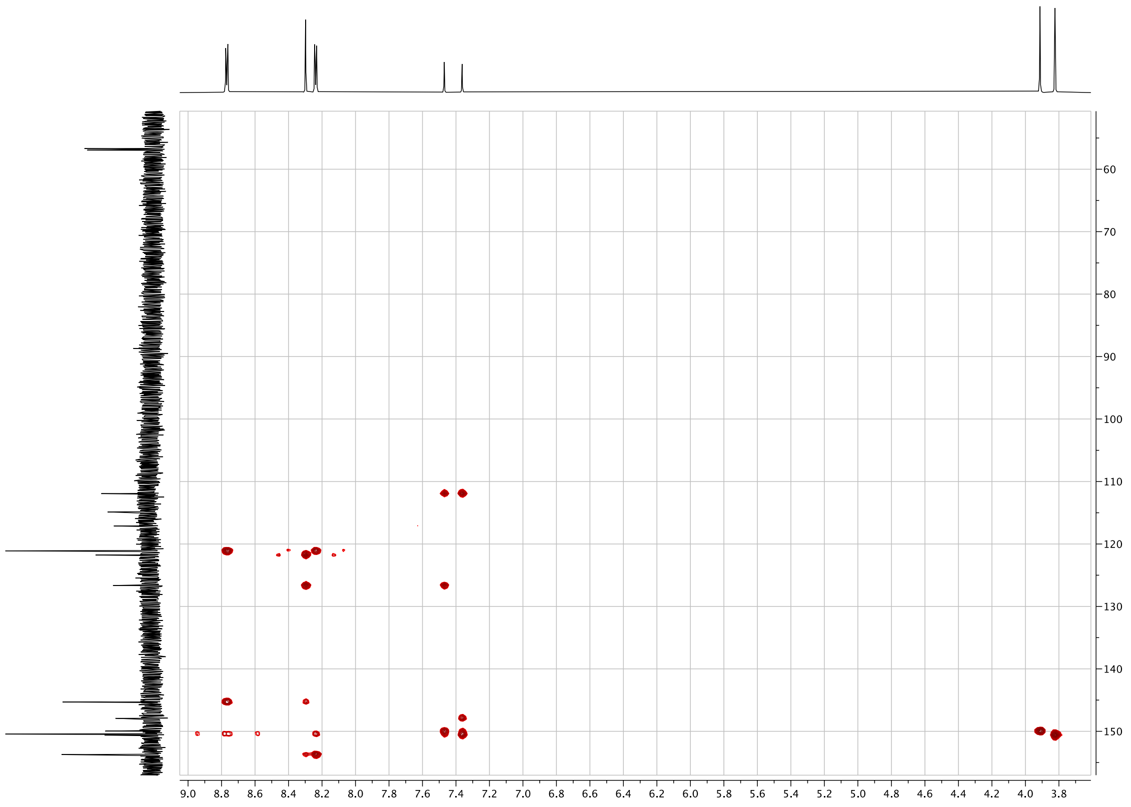

Figure S10. HMBC spectrum of **4** (^{1}H 500 MHz, ^{13}C 126 MHz, DMSO-d_6, 298 K).

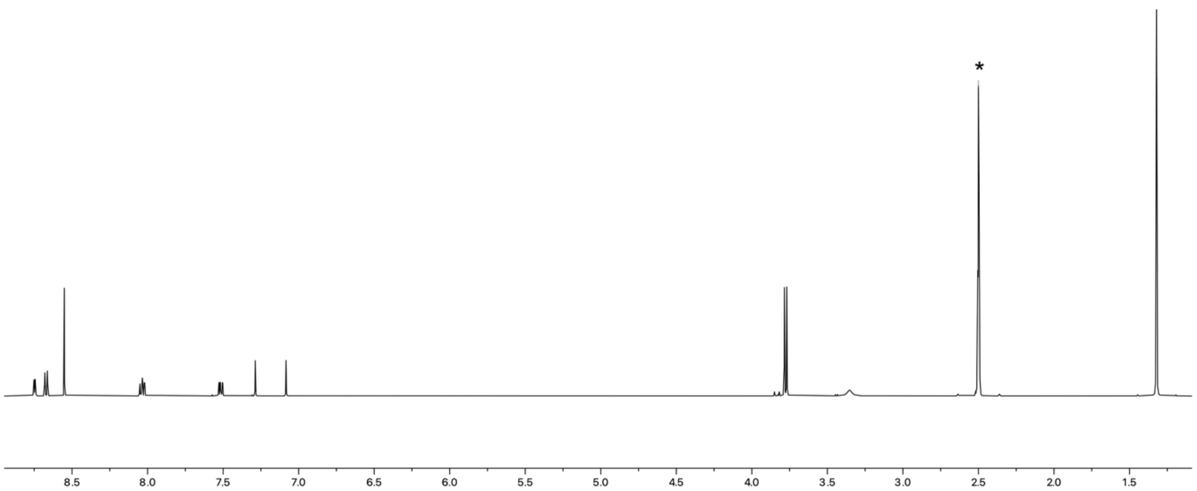

Figure S11. ^{1}H NMR spectrum of **2** (^{1}H 500 MHz DMSO-d_6, 298 K). * = residual DMSO-d_5.

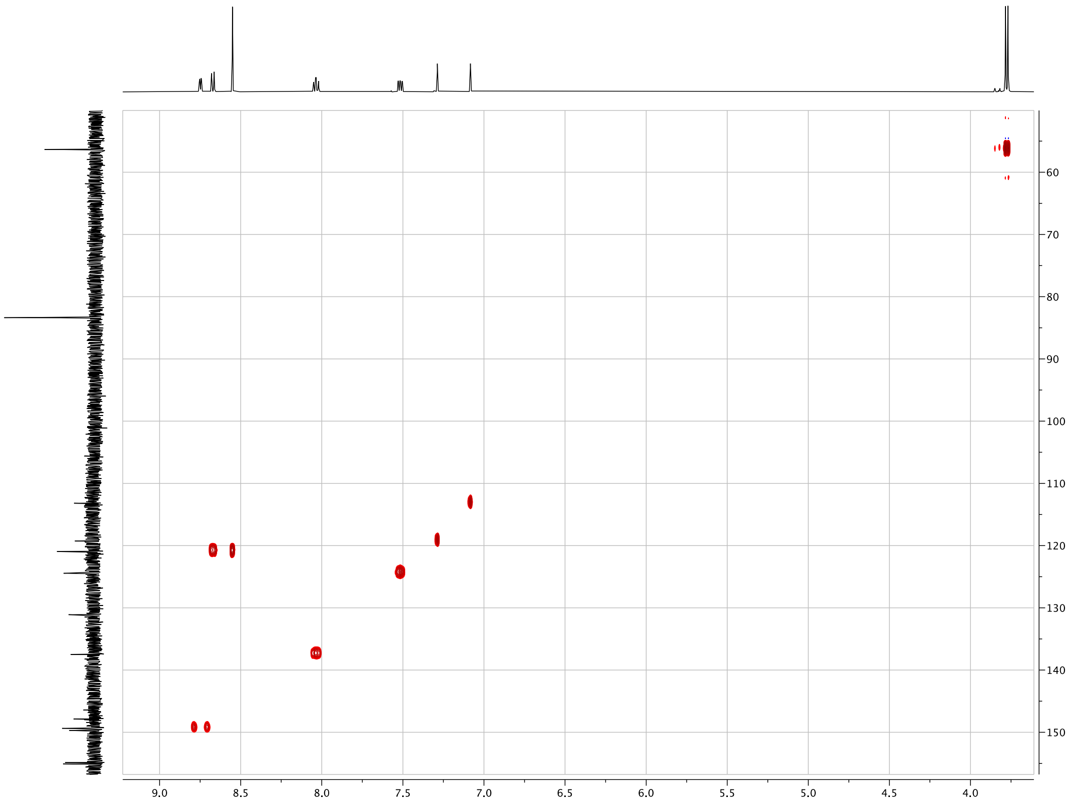

Figure S12. HMQC spectrum of **2** (^{1}H 500 MHz, ^{13}C 126 MHz, DMSO-d_6, 298 K).

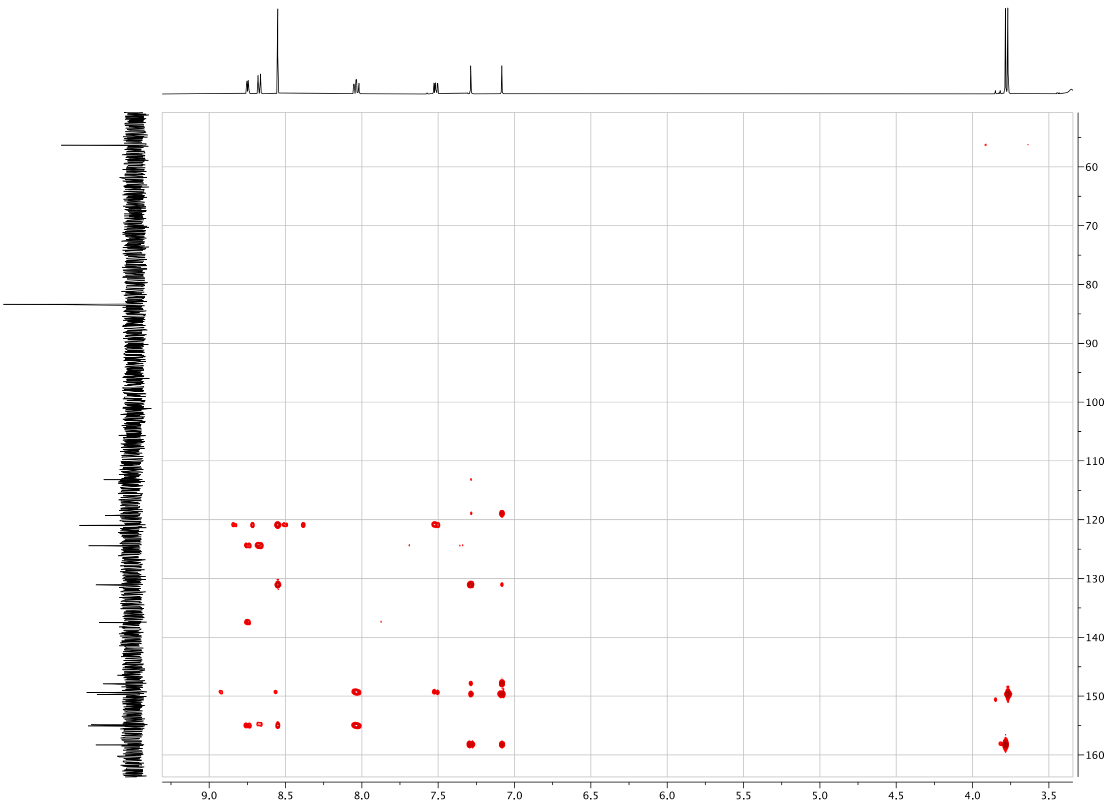

Figure S13. HMBC spectrum of **2** (^{1}H 500 MHz, ^{13}C 126 MHz, DMSO-d_6, 298 K).

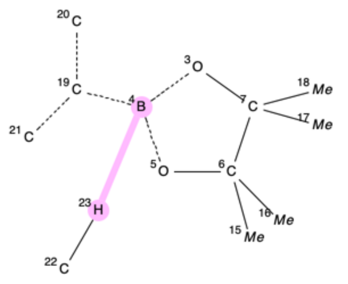

Figure S14. The search motif used in Conquest (version 2022.2.0) [1]. Both B and C(19) were defined as 3-coordinate; --- = any bond type; normalized H coordinates were applied. The H...B contact was defined as ≤ sum of the van der Waals radii.

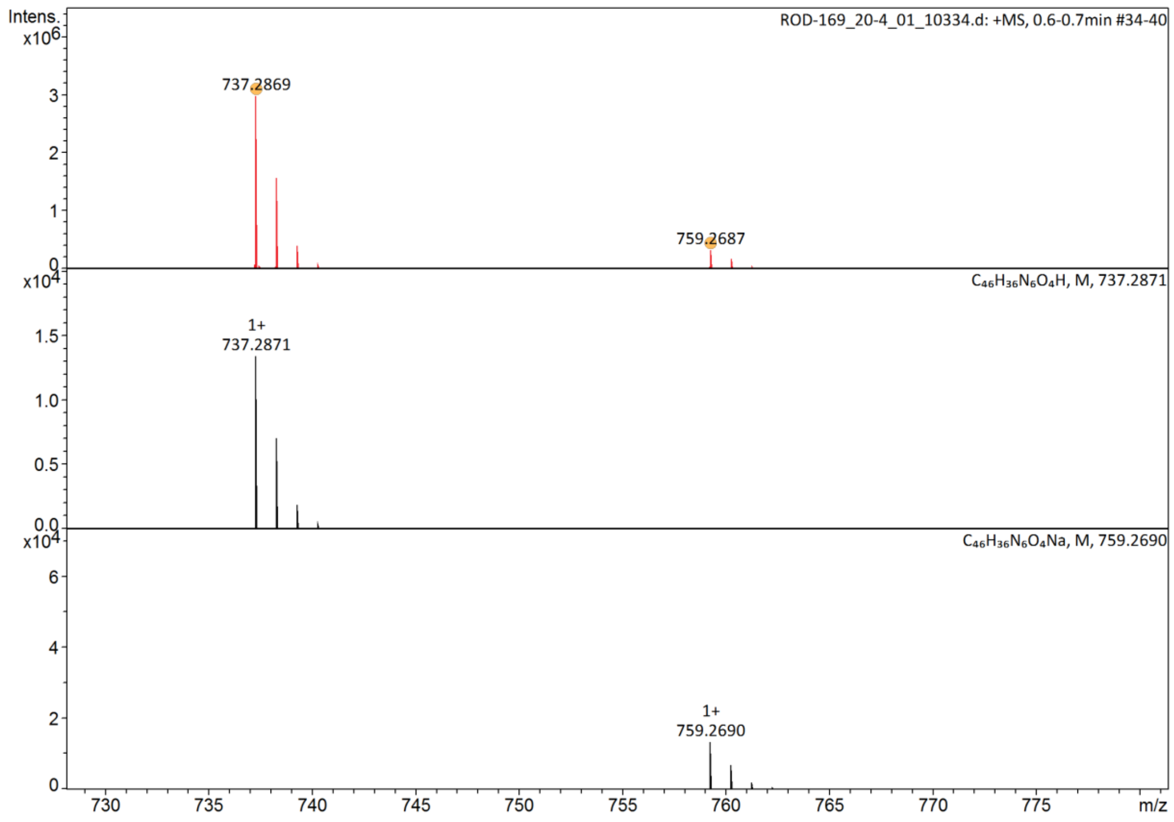

Figure S15. The [M+H]$^+$ and [M+Na]$^+$ peaks in the high resolution electrospray mass spectrum of **5**. Top, experimental; bottom, simulated.

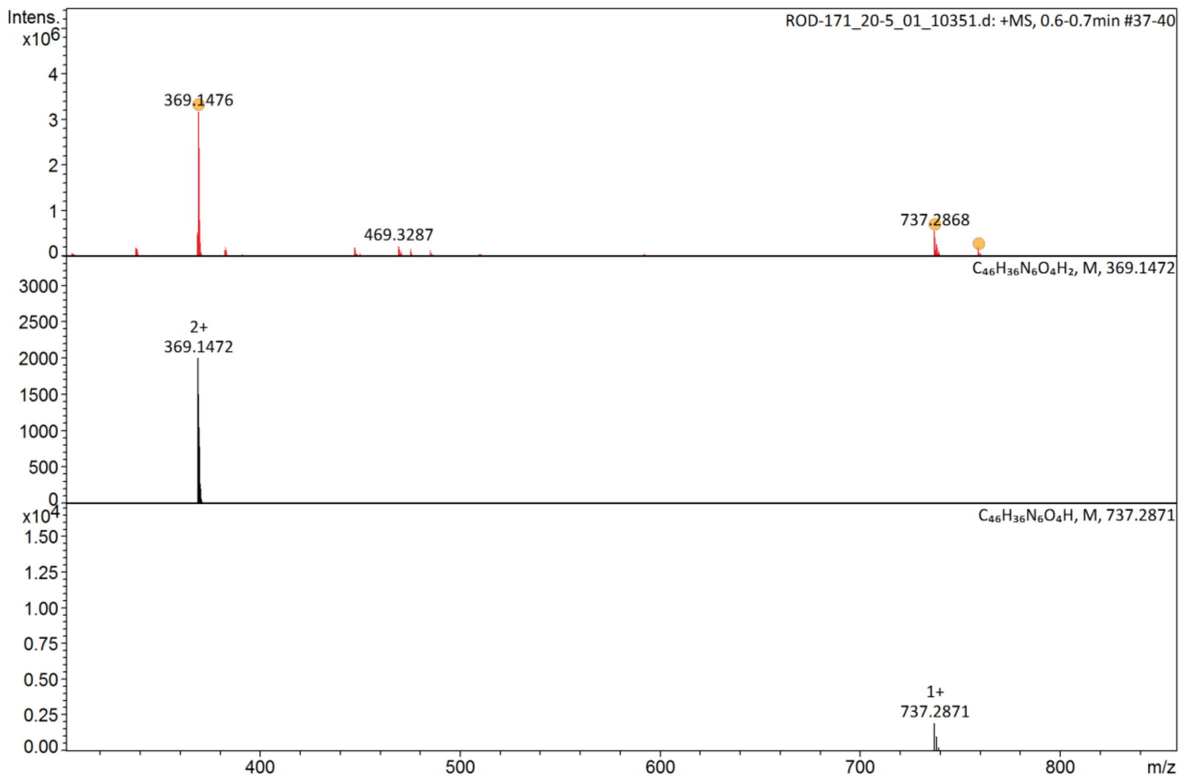

Figure S16. The [M+2H]$^{2+}$ and [M+H]$^+$ peaks in the high resolution electrospray mass spectrum of **6**. Top, experimental; bottom, simulated.

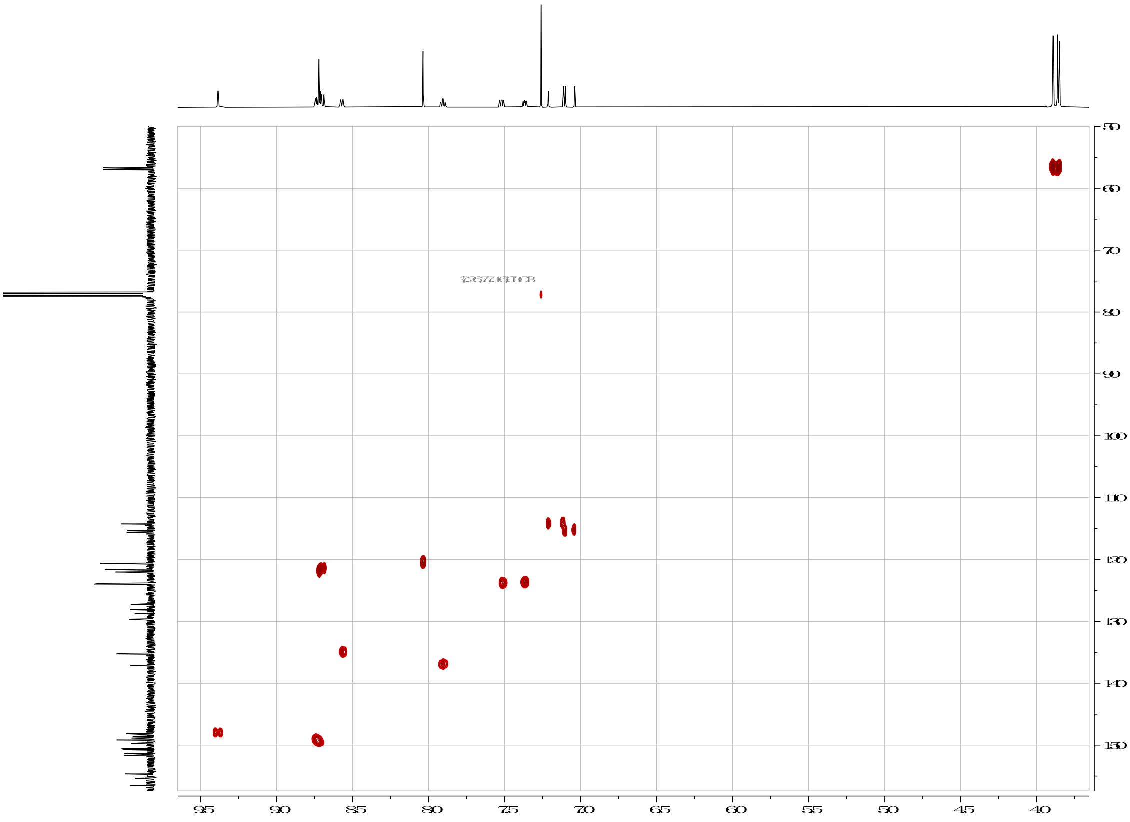

Figure S17. HMQC spectrum of **5** (^{1}H 500 MHz, ^{13}C 126 MHz, $CDCl_3$, 298 K).

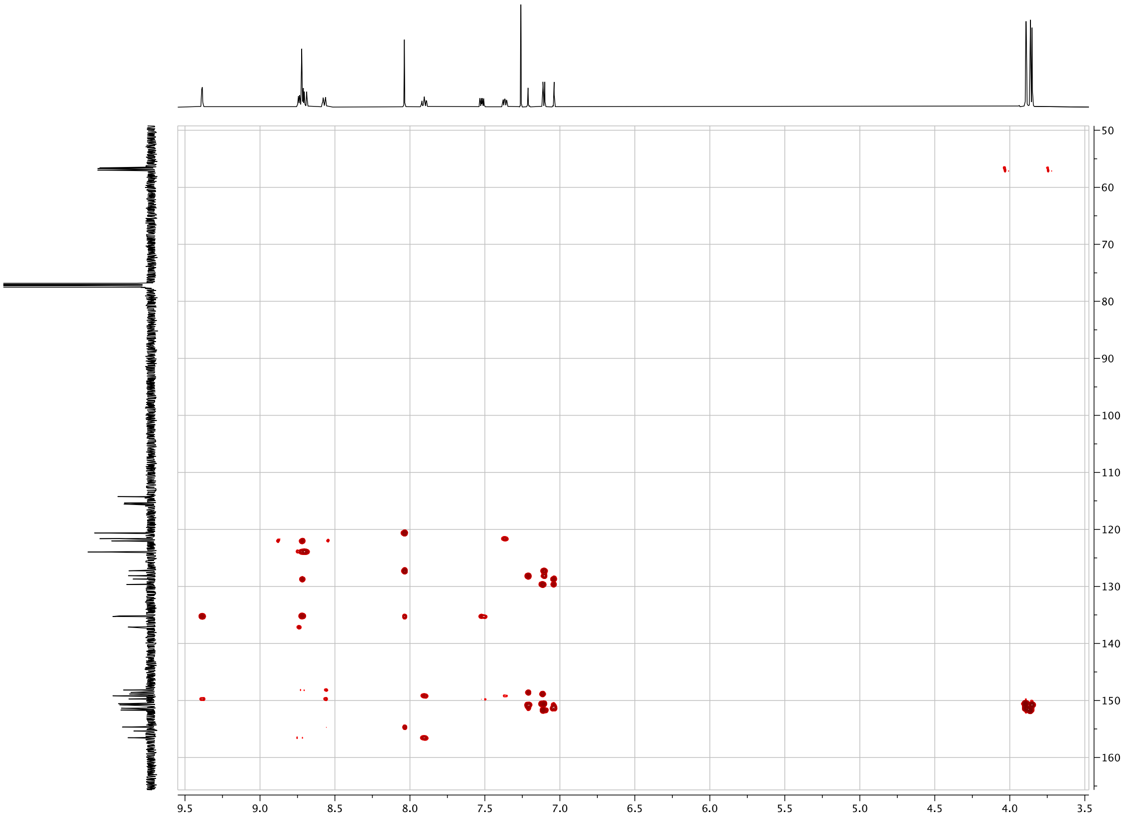

Figure S18. HMBC spectrum of **5** (^{1}H 500 MHz, ^{13}C 126 MHz, $CDCl_3$, 298 K).

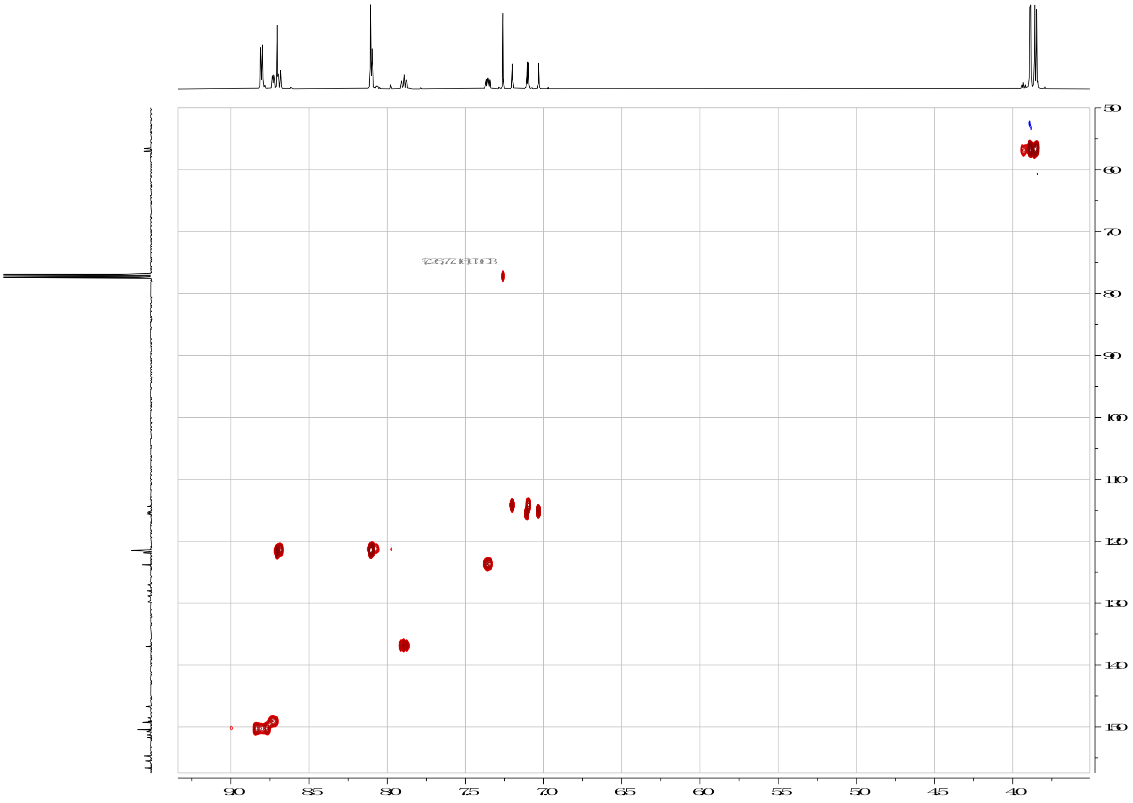

Figure S19. HMQC spectrum of **6** (^{1}H 500 MHz, ^{13}C 126 MHz, $CDCl_3$, 298 K).

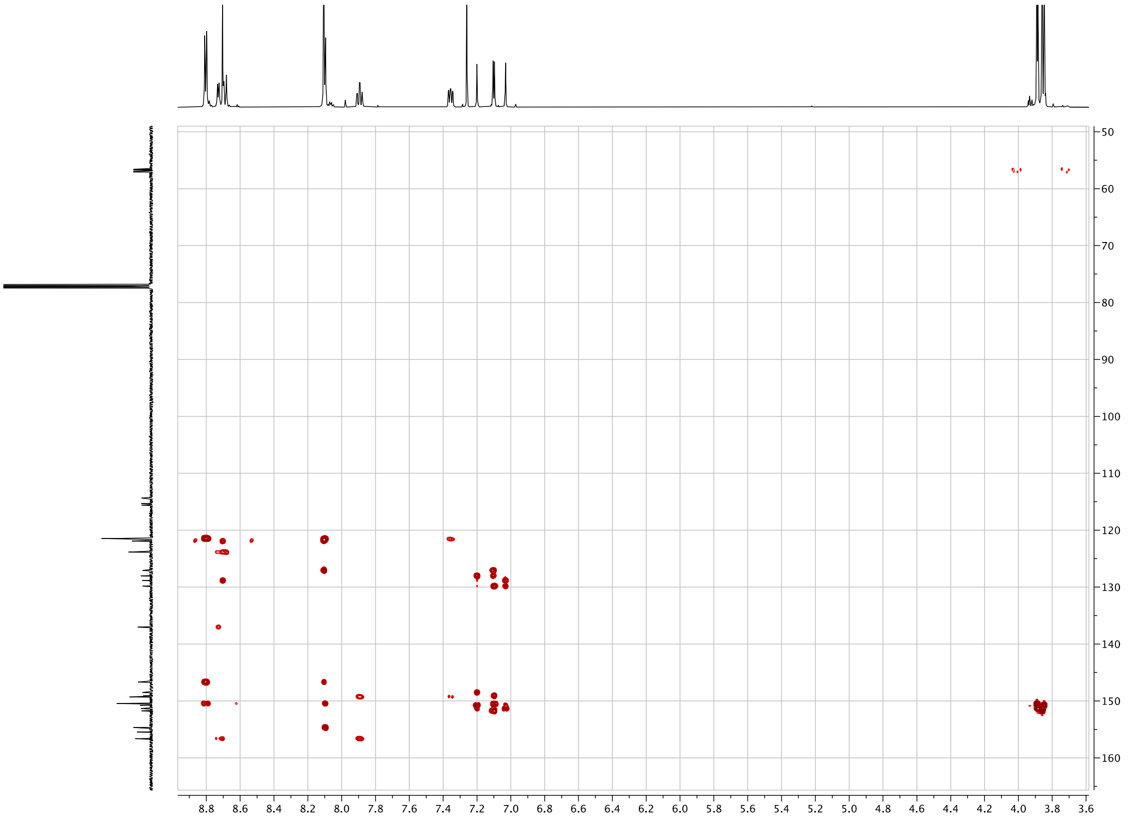

Figure S20. HMBC spectrum of **6** (^{1}H 500 MHz, ^{13}C 126 MHz, $CDCl_3$, 298 K).

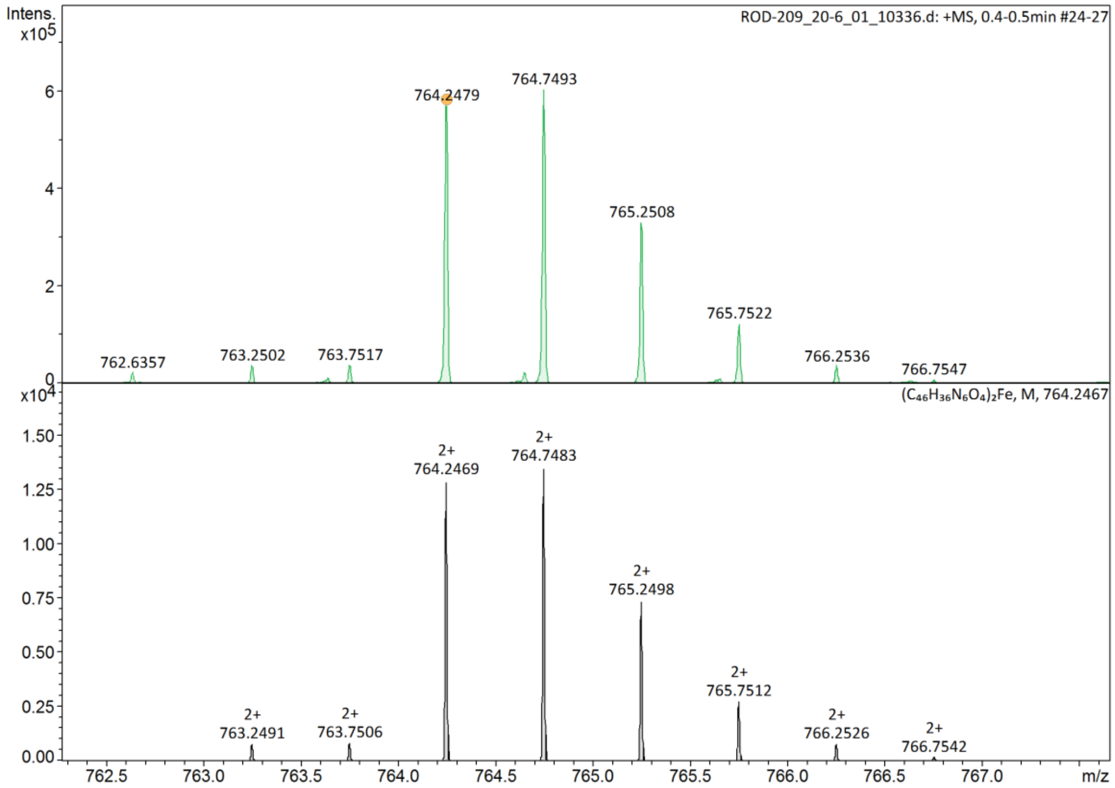

Figure S21. The $[M]^{2+}$ peak in the high resolution electrospray mass spectrum of $[Fe(\mathbf{5})_2][NO_3]_2$: top, experimental spectrum; bottom, simulated spectrum.

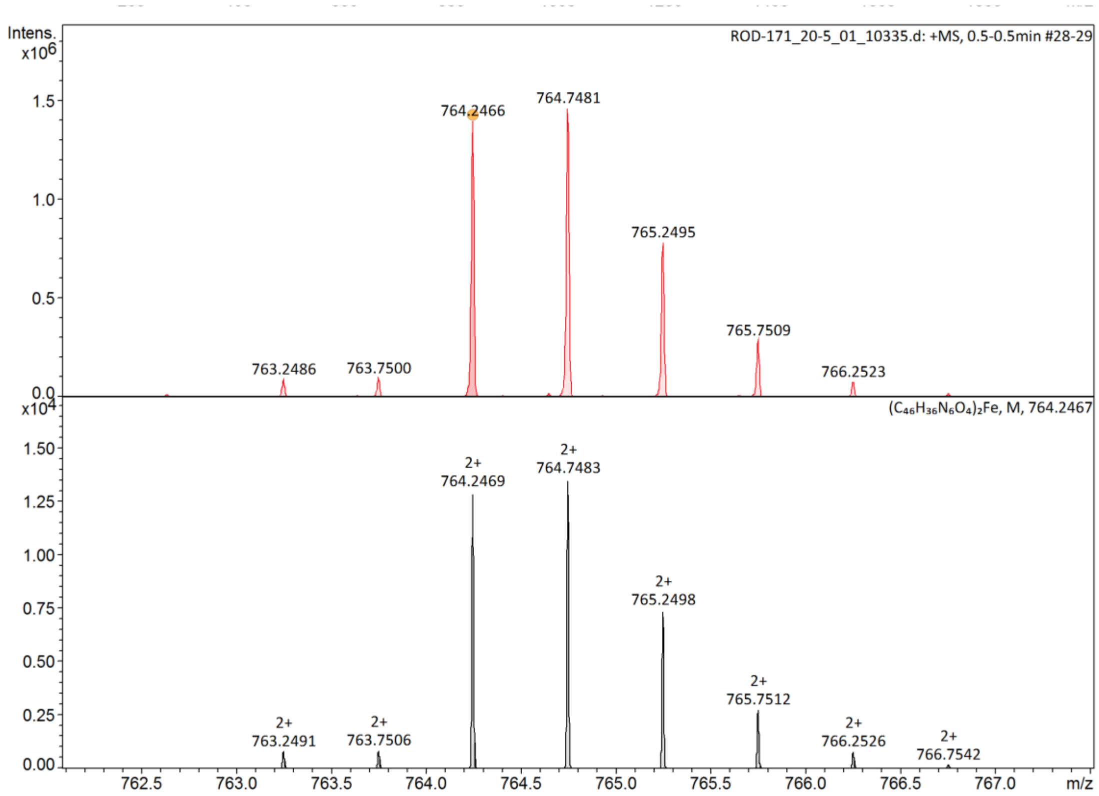

Figure S22. The $[M]^{2+}$ peak in the high resolution electrospray mass spectrum of $[Fe(\mathbf{6})_2][BF_4]_2$: top, experimental spectrum; bottom, simulated spectrum.

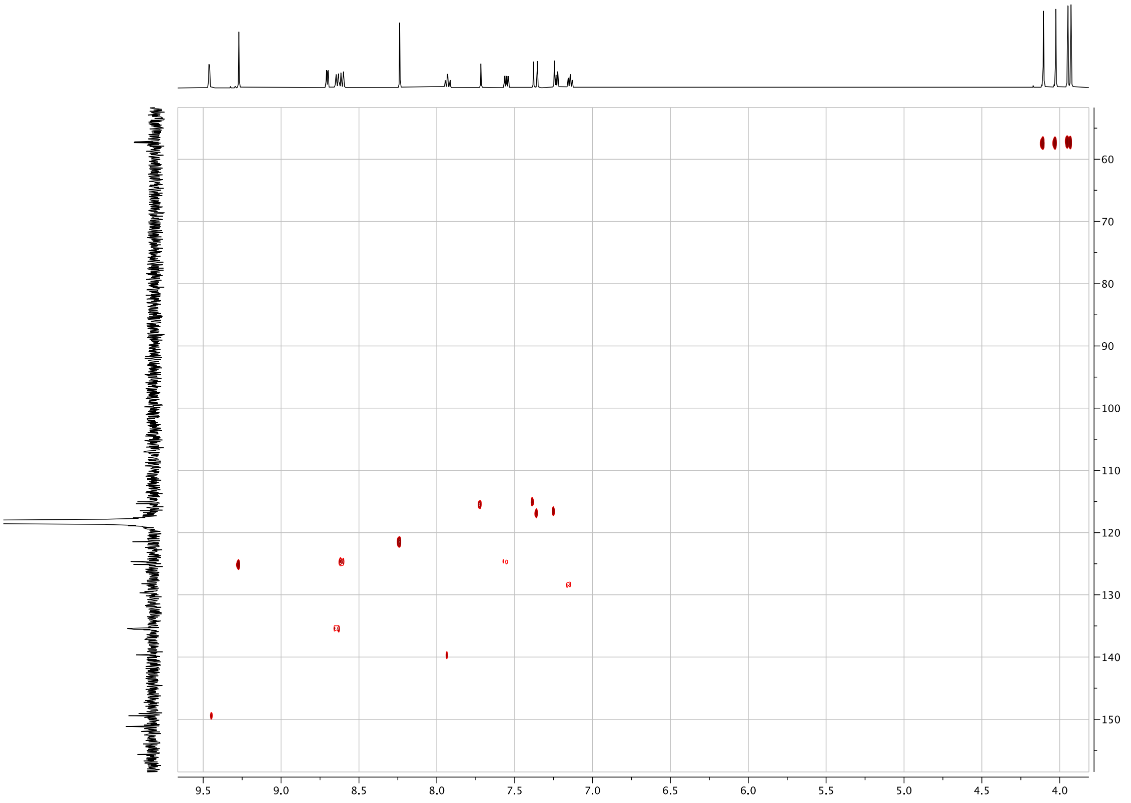

Figure S23. HMQC spectrum of [Fe(**5**)$_2$][NO_3]$_2$ (^{1}H 500 MHz, ^{13}C 126 MHz, CD_3CN, 298 K).

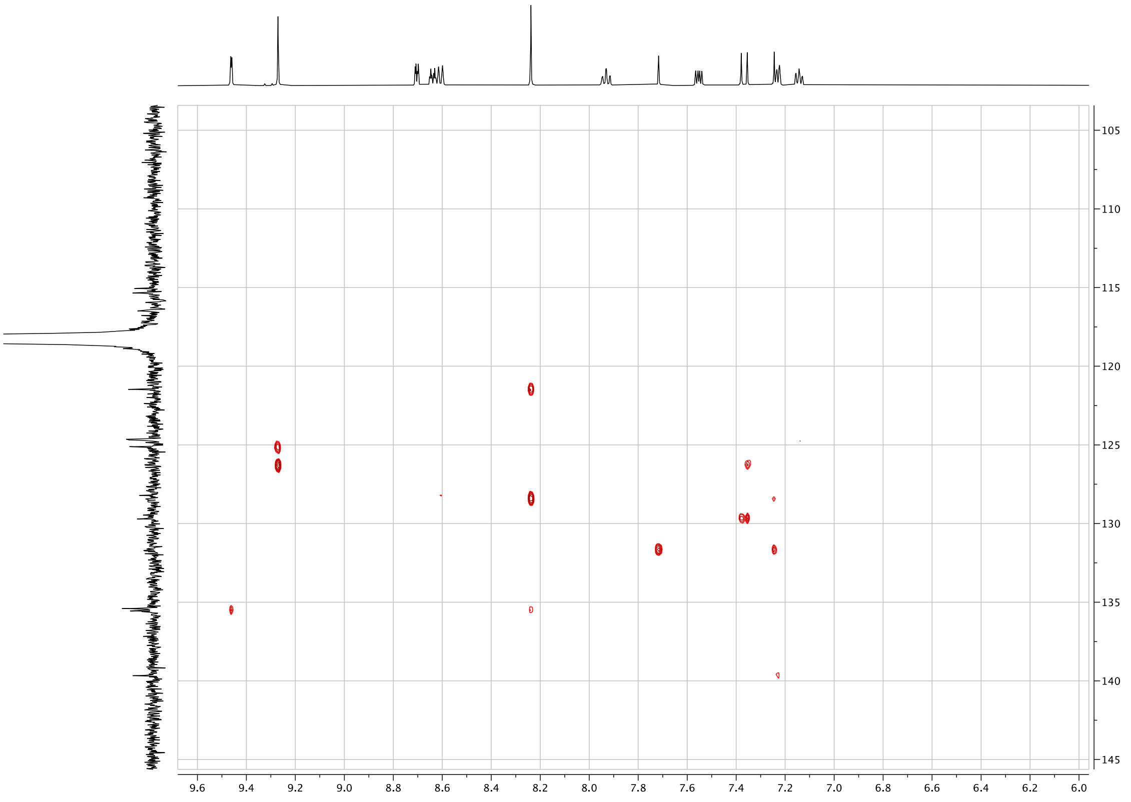

Figure S24. HMBC spectrum of [Fe(**5**)$_2$][NO_3]$_2$ (^{1}H 500 MHz, ^{13}C 126 MHz, CD_3CN, 298 K).

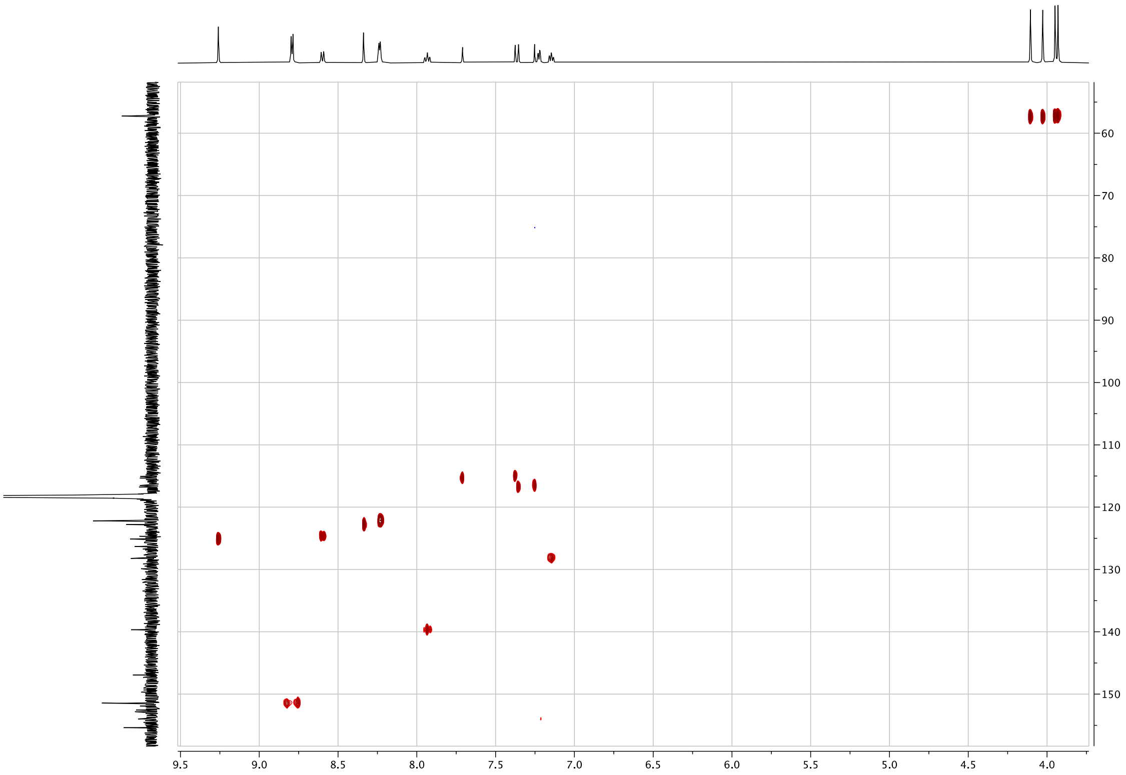

Figure S25. HMQC spectrum of [Fe(**6**)$_2$][BF$_4$]$_2$ (^{1}H 500 MHz, ^{13}C 126 MHz, CD_3CN, 298 K). ^{13}C{^{1}H} signal at d 118.26 ppm arises from CD_3CN.

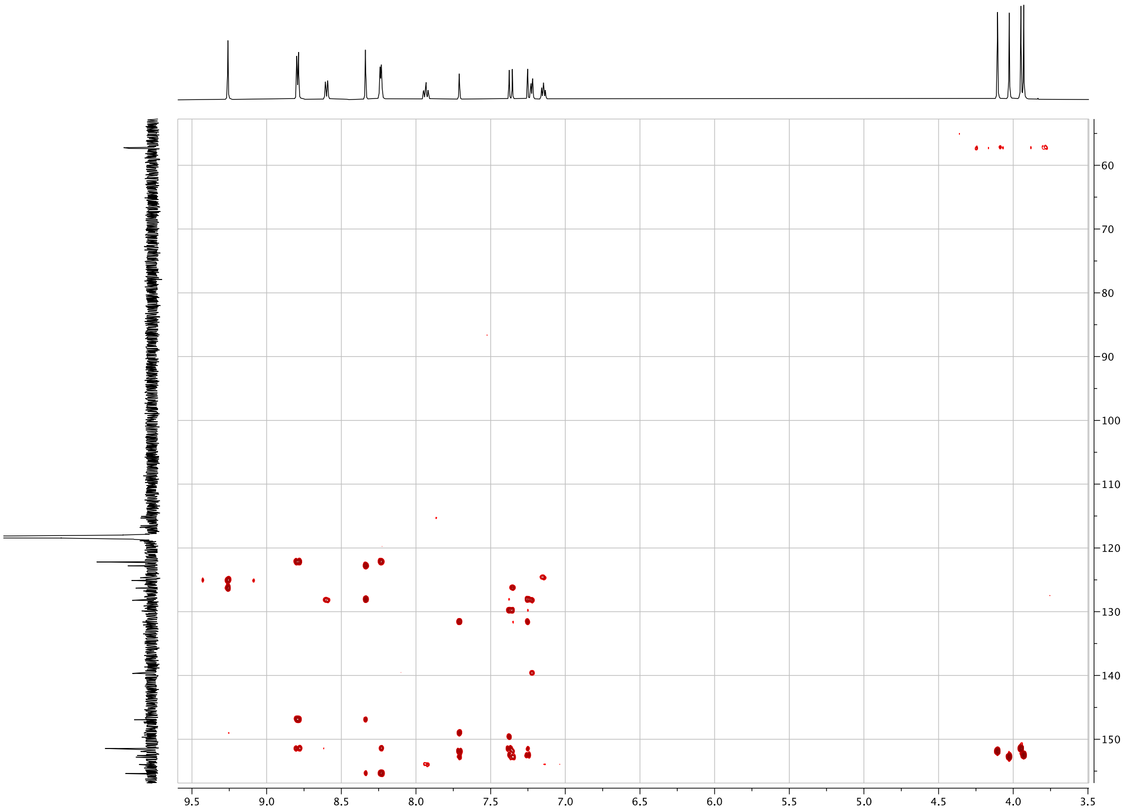

Figure S26. HMBC spectrum of $[Fe(\mathbf{6})_2][BF_4]_2$ (^{1}H 500 MHz, ^{13}C 126 MHz, CD_3CN, 298 K). $^{13}C\{^1H\}$ signal at d 118.26 ppm arises from CD_3CN.

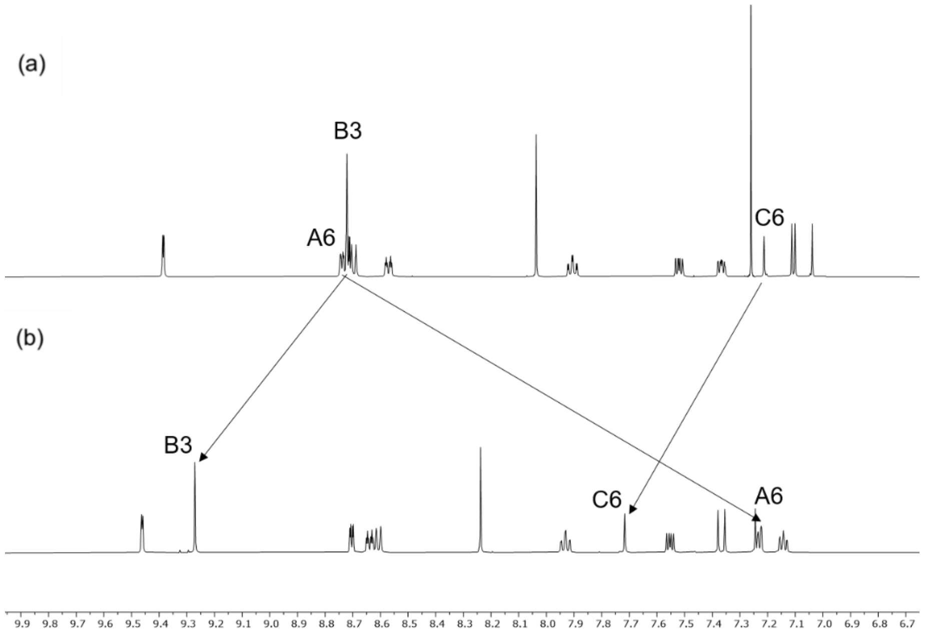

Figure S27. Comparison of the aromatic regions of the ^{1}H NMR spectra (500 MHz, 298 K) of (a) compound **5** in $CDCl_3$, and (b) $[Fe(\mathbf{5})_2][NO_3]_2$ in CD_3CN. The effect of coordination on the chemical shifts of the signals for protons H^{A6}, H^{B3} and H^{C6} (see Scheme 5 for atom labels) is highlighted.

Reference

1. Bruno, I.J.; Cole, J.C.; Edgington, P.R.; Kessler, M.; Macrae, C.F.; McCabe, P.; Pearson, J.; Taylor, R. New software for searching the Cambridge Structural Database and visualising crystal structures. *Acta Cryst.* **2002**, *B58*, 389-397. doi: 10.1107/S0108768102003324
